# Hormone replacement therapy (conjugated oestrogens plus bazedoxifene) for post-menopausal women with symptomatic hand osteoarthritis: primary report from the HOPE-e randomised, placebo-controlled, feasibility study

**DOI:** 10.1016/S2665-9913(22)00218-1

**Published:** 2022-09-21

**Authors:** Jennifer A E Williams, Mae Chester-Jones, Catherine Minns Lowe, Megan V Goff, Anne Francis, Gretchen Brewer, Ioana Marian, Susan L Morris, Debbie Warwick, Lucy Eldridge, Patrick Julier, Malvika Gulati, Karen L Barker, Vicki S Barber, Joanna Black, Sue Woollacott, Charles Mackworth-Young, Vicki Glover, Sarah E Lamb, Tonia L Vincent, Katy Vincent, Susan J Dutton, Fiona E Watt

**Affiliations:** a Oxford Clinical Trials Research Unit (OCTRU), Nuffield Department of Orthopaedics, Rheumatology and Musculoskeletal Sciences (NDORMS), University of Oxford, Oxford, UK; b Centre for Osteoarthritis Pathogenesis Versus Arthritis, Kennedy Institute of Rheumatology, NDORMS, University of Oxford, Oxford, UK; c OCTRU, Centre for Statistics in Medicine, NDORMS, University of Oxford, Oxford, UK; d Physiotherapy Research Unit, Physiotherapy Department, Nuffield Orthopaedic Centre, Oxford University Hospitals NHS Foundation Trust, Oxford, UK; e Cancer Prevention Trials Unit, King's College London Guy's Campus, London, UK; f National Institute for Health and Care Research Clinical research Network (Thames Valley and South Midlands), Nuffield Orthopaedic Centre, Oxford University Hospitals NHS Foundation Trust, Oxford, UK; g White Horse Medical Practice, Faringdon, UK; h Rheumatology Department, Charing Cross Hospital, Imperial College Healthcare NHS Trust, London, UK; i Centre for Rehabilitation Research, NDORMS, Oxford, University of Oxford, UK; j College of Medicine and Health, University of Exeter, Exeter, UK; k Nuffield Department of Women's and Reproductive Health, University of Oxford, Oxford, UK; l Centre for Inflammatory Disease, Department of Immunology and Inflammation, Imperial College London, London, UK

## Abstract

**Background:**

Symptomatic hand osteoarthritis is more common in women than in men, and its incidence increases around the age of menopause, implicating oestrogen deficiency. No randomised controlled trials of hormone replacement therapy (HRT) have been done in people with hand osteoarthritis. We aimed to determine the feasibility and acceptability of a form of HRT (conjugated oestrogens plus bazedoxifene) in post-menopausal women with painful hand osteoarthritis.

**Methods:**

The HOPE-e feasibility study was a randomised, double-blind, placebo-controlled trial, for which we recruited women aged 40–65 years, for whom 1–10 years had passed after their final menstrual period, with definite hand osteoarthritis and at least two painful hand joints. Participants were recruited across three primary or secondary care sites and from the community and were randomly assigned (1:1) to receive conjugated oestrogens plus bazedoxifene or placebo, orally once every day for 24 weeks, before weaning for 4 weeks until the end of the study. The primary feasibility outcomes were rates of identification, recruitment, randomisation, retention, and compliance of eligible participants, and the likelihood of unmasking. The secondary objective was to generate proof-of-concept quantitative and qualitative data on the acceptability of proposed clinical outcomes for a full trial and adverse events. We used an intention-to-treat analysis, and criteria for progression to a full trial were pre-defined as recruitment of at least 30 participants across all sites in 18 months; a dropout rate of less than or equal to 30% of randomised individuals; and acceptability to the majority of participants, including acceptable rates of adverse events. Due to the COVID-19 pandemic, the recruitment window was reduced to 12–15 months. A proportionately reduced minimum sample size of 22 was judged to be sufficient to test feasibility. This trial was registered at ISRCTN, ISRCTN12196200.

**Findings:**

From May 9, 2019 to Dec 31, 2020, 434 enquiries or referrals were received. We did 96 telephone pre-screens; of the 35 eligible participants, seven were excluded as ineligible at the telephone or face-to-face screening and 28 (80% [95% CI 63–92]) were randomly assigned. Of the 406 who were not randomly assigned, 250 (62%) were ineligible (with contraindicated medications accounting for 50 [20%] of these), 101 (25%) did not respond to further enquiries, and 55 (14%) chose not to proceed (with the most common reason being not wanting to take a hormone-based drug). All 28 randomised participants completed all follow-up assessments with high compliance and outcome measure completeness. All three adverse event-related treatment withdrawals were in the placebo group. No serious adverse events were reported. Participants and investigators were successfully masked (participant Bang's blinding index placebo group 0·50 [95% CI 0·25–0·75]). The trial met the prespecified criteria for progression to a full trial.

**Interpretation:**

This first-ever feasibility study of a randomised controlled trial of HRT for post-menopausal women with painful hand osteoarthritis met its progression criteria, although it was not powered to detect a clinical effect. This outcome indicates that a full trial of an HRT in this population is feasible and acceptable and identifies potential refinements with regard to the design of such a trial.

**Funding:**

Research for Patient Benefit programme, National Institute for Health Research.

## Introduction

Osteoarthritis is the most common form of arthropathy worldwide and has been recently designated as a serious disease by the US Food and Drug Administration.^1^ Hand osteoarthritis is one of its most prevalent forms, affecting 40% of people over their lifetime.^2^ For many, it causes unacceptable levels of pain, dysfunction, and reduced quality of life similar to rheumatoid arthritis.^2^, ^3^

The female preponderance of osteoarthritis in general, but in particular for symptomatic or erosive hand osteoarthritis, is striking.^4^ The incidence of symptomatic hand osteoarthritis in women is more than twice that in men, with a notable increase around the age of 50 years (the median age of menopause worldwide).^5^ Menopause is a period of variable and increasing oestrogen deficiency. The term arthritis of the menopause was coined as long ago as the 1920s for peri-menopausal or menopausal women presenting with generalised osteoarthritis, including hand osteoarthritis.^6^ There is compelling evidence from animal models for the importance of oestrogen in the development of knee osteoarthritis, but no animal models of hand osteoarthritis exist, making mechanistic studies difficult.^7^, ^8^

**Research in context**

**Evidence before this study**

Novel pharmacological agents for osteoarthritis that improve joint pain and function are a high research priority. Female sex is a well-established risk factor for symptomatic osteoarthritis, particularly hand osteoarthritis. Hand osteoarthritis is most common in women during the decade associated with menopause, a time of changes in sex hormones. Musculoskeletal symptoms, such as joint pain, are frequently a part of menopause syndrome. However, there are only sparse musculoskeletal data available from large hormone replacement therapy (HRT) trials. The Women's Health Initiative recruited participants with classic menopausal symptoms. Those on unconjugated oestrogens were more protected from large joint replacement and incident musculoskeletal pain than were those on placebo. Before planning this study, we searched PubMed for articles published in English reporting cohort studies, observational or controlled studies, or randomised controlled trials relating to the effects of HRT agents in people with osteoarthritis, using the terms [“osteoarthritis” OR “hand osteoarthritis”] AND [“HRT” OR “Hormone” OR “Hormone Replacement Therapy”] OR [“estrogen” OR “oestrogen” OR “estradiol”] OR [“selective estrogen receptor modulator” OR “SERM”] from inception to Jan 1, 2016. We found no relevant studies or randomised controlled trials, or any reports of feasibility or acceptability assessments of this drug class in people with osteoarthritis. The only relevant article was a meta-analysis by Sniekers and colleagues, which corroborated the protective effect of oestrogen on hip arthroplasty in large trial populations but also noted some risk of bias.

**Added value of this study**

This study shows that running a clinical trial of a form of HRT in post-menopausal women with painful hand osteoarthritis is feasible and acceptable to those taking part. This information is crucial given the often contentious nature of HRT and its safety considerations, both real and perceived. The study was not designed to test the efficacy or safety of HRT in this population and so cannot and must not change practice. However, it provides feasibility and proof-of-concept data for the first time in women with moderate-to-high symptoms of hand osteoarthritis, who were randomly assigned to drug or placebo. The types and frequencies of safety events were similar to other HRT trials (probably because the population characteristics were very similar). Because of biases inherent to use of observational or electronic health-care data, such evidence is crucial for answering remaining questions. Some symptoms in the active treatment group appeared to get worse when the drug was stopped, which supports similar observations from electronic health-care record studies. If cessation of HRT causes a rebound in musculoskeletal or other symptoms, understanding the best way to wean and stop HRT to prevent flare-up of existing or new issues should be identified. This study also highlights methods for recruiting to osteoarthritis studies beyond the COVID-19 pandemic, such as remote assessment. Recruiting from the community increases numbers and generalisability. A low-cost, real-time way of measuring mean hand pain was acceptable and provided high-quality data (comparable to recalled pain), supporting its use in studies of this kind.

**Implications of all the available evidence**

Post-menopausal women with symptomatic hand osteoarthritis might represent an important disease subtype. There is evidence that painful musculoskeletal conditions might start or get worse around the time of menopause or cessation of HRT—the high interest and enquiries for this study support this conclusion, which should be considered when taking a history of either a painful musculoskeletal condition or symptoms of menopause. Our study does not provide evidence for effect but does support the need to understand whether there might be sex-specific treatments or treatment considerations for people with rheumatic diseases. It also reinforces the importance of understanding the mechanisms by which oestrogen deficiency or other sex hormone changes might contribute to the risk of illness or disease.

There is an ongoing unmet need for effective drug therapy for pain or disease modification in patients with hand osteoarthritis: a series of clinical trials failed to show efficacy in repurposing a range of anti-rheumatic drugs for people with hand osteoarthritis, suggesting that novel approaches are needed that account for disease-specific mechanisms.^9^, ^10^, ^11^ There is a recognition, as in other areas of rheumatology, that clinically relevant subgroups of hand osteoarthritis are likely to exist and that stratification might help to identify effective interventions.^12^ Therefore, the question of whether treating oestrogen deficiency with hormone replacement therapy (HRT) could improve or prevent osteoarthritis symptoms or structural joint changes in humans is relevant.

In a trial run as part of the Women's Health Initiative, people receiving unconjugated oestrogens had lower rates of hip and knee arthroplasty and less all-cause musculoskeletal pain than those receiving placebo in post-hoc studies.^13^, ^14^ More recently, both small and large UK health record datasets have suggested that the use of HRT might influence the onset of symptoms of hand osteoarthritis.^15^ However, no causal associations can be inferred from these studies and there are inherent biases related to the reasons women might seek HRT (50% of menopausal women have related musculoskeletal symptoms^16^). Selective oestrogen receptor modulators (a newer class of HRT drug) have been shown in pre-clinical models to have beneficial effects on articular cartilage and have been associated with improvements in musculoskeletal pain.^17^, ^18^, ^19^ A first-in-class combination of a selective oestrogen receptor modulator and unconjugated oestrogens is licensed for use for menopausal symptoms.^20^, ^21^ Currently, the use of HRTs for a primary indication of musculoskeletal symptoms is outside of the license for these therapies.

To our knowledge, no randomised controlled trials have ever been done to test the efficacy of an HRT agent on joint pain in patients with symptomatic hand osteoarthritis.^16^ There are safety considerations around the use of HRT, which raise the question of acceptability and feasibility of use of this class of agent in people with hand osteoarthritis. Hesitancy among both clinicians and society still exists in the wake of the Women's Health Initiative, despite growing high-quality evidence that HRT is a reasonable option for many women with uncontrolled symptoms of menopause. Safety is a consideration, but vascular and cancer risks are minimised when used in the first 5–10 years after the final menstrual period, applying appropriate medical exclusions and using newer agents.^22^, ^23^ For these reasons, we aimed to test the feasibility and acceptability of use of an HRT consisting of a selective oestrogen receptor modulator plus conjugated oestrogens in post-menopausal women with painful hand osteoarthritis, and to generate proof-of-concept data in a randomised, placebo-controlled, feasibility study that could enable design of a subsequent full trial if the progression criteria were met.

## Methods

### Study design and participants

The HOPE-e feasibility study was a randomised, double-blind, placebo-controlled trial of an oral HRT in post-menopausal women with symptomatic hand osteoarthritis. There were three study sites in England (Nuffield Orthopaedic Centre, Oxford University Hospitals NHS Foundation Trust, Oxford, UK; Charing Cross Hospital, Imperial College Healthcare NHS Trust, London, UK; and White Horse Medical Practice, Faringdon, UK), across primary and secondary care. Individuals were recruited from these study sites and also from the community. To be eligible, participants had to be women aged 40–65 years, for whom 1–10 years had passed since their final menstrual period, with hand osteoarthritis that fulfilled the American College of Rheumatology clinical diagnostic criteria.^24^ Participants also had to have pain in at least two hand joints (reported typically as at least 4 on a scale of 10), despite trying core guidance for the management of hand osteoarthritis.^25^ Patients with other causes of hand pain, current or recent use of prohibited medications, or a medical contraindication to the use of systemic HRT were excluded.^22^, ^23^ Participants who were taking other medications for hand pain needed to be on a stable dose for at least 4 weeks before starting the study medication. These participants were asked to continue these medications at their current dose and to avoid starting new medications that could influence pain during the study. When not already available in the past 3 years and clinically indicated, a radiograph of both hands was performed to support diagnosis as part of usual care. Full eligibility criteria are shown in the appendix (pp 5–6). Due to the COVID-19 pandemic, the recruitment window was reduced to 15 months. The minimum recruitment target was adjusted proportionately, and this was judged to be still sufficient to test feasibility. Written informed consent was given by all participants. Ethical approval was issued by the UK North of Scotland Research Ethics Committee 2 (reference 18/NS/0100). The protocol has been published previously.^26^ Amendments that were made to the protocol after publication are detailed in the appendix (pp 2–4). Patient and public involvement began ahead of funding, with a patient discussion group and lay co-applicant actively involved in the rationale, design, and development of the protocol and patient-facing materials. There were also two people with lived experience of osteoarthritis on the Trial Steering Committee.

### Randomisation and masking

Participants were randomly allocated 1:1 to receive conjugated oestrogens (0·45 mg) plus bazedoxifene acetate (20 mg; Pfizer; Sandwich, UK) or placebo via a web-based service maintained by the Oxford Clinical Trials Research Unit, using a minimisation algorithm that included a random element. Participants were stratified by study site and clinical subtype of painful joints (ie, interphalangeal, base of thumb, or both). Participants, site staff (except pharmacy staff), and patient-facing central study staff were all masked to treatment allocation and any intentional or unintentional unmasking was recorded. Further details can be found in the protocol.^26^

### Procedures

The conjugated oestrogen–bazedoxifene combination was purchased from Pfizer (Sandwich, UK), via the UK National Health Service (NHS) Supply Chain. A blistered, closely matched placebo was manufactured by Modepharma (Beckenham, UK). Both agents were overpackaged at dispensing to maintain allocation concealment. Participants took conjugated oestrogen plus bazedoxifene or matched placebo once daily as an oral tablet for 24 weeks, from the baseline visit to the week 24 visit; participants were then weaned gradually and stopped the medication over a further 4 weeks (a weaning period was included on the basis of typical clinical practice when stopping an HRT, to try to avoid symptom flare if there had been benefit^27^). Follow-up visits were carried out at weeks 4, 12, 24, and 28 (appendix pp 7–8). Participants were also invited to attend one of two optional focus groups to explore acceptability at the end of the study (appendix p 29).

### Outcomes

The primary objective was to assess the feasibility of a fully powered, randomised, controlled trial. The secondary objectives were to generate proof-of-concept data, refine the methodology, and determine the acceptability of the study design and treatment. The primary outcomes to determine feasibility were recruitment and randomisation rates from different recruitment sources; retention rates; study medication compliance (participant-reported adherence, assessed via daily recording in diaries); and likelihood of unintentional unmasking, assessed using Bang's blinding index.^28^ Secondary outcomes (provisional outcomes for a full trial^26^) included patient-reported mean hand pain during the 14 days before each visit (which would be the primary outcome for a full trial).^29^ This outcome was measured by two methods on a scale of 0–10, for which 0 indicated no pain and 10 indicated the worst pain imaginable: (1) by recall at visits, and (2) by remote daily rating via paper or electronically via weblink in a text message (with a calculated mean of a minimum of 3 to a maximum of 14 scores in the intention-to-treat population). Other secondary outcomes were: investigator-recorded, participant-reported individual painful joint count; hand function (assessed with the Functional Index for Hand Osteoarthritis [FIHOA] questionnaire^29^ and by average grip strength in each hand as measured by a dynamometer); hand appearance (patient-reported cosmesis score from Michigan Hand Questionnaire and hand photographs); investigator-recorded tender and swollen joint count; menopausal symptoms and quality of life (Menopause-Specific Quality Of Life [MENQOL]^21^ questionnaire [intervention 1-month recall version] and Greene Climacteric Scale [GCS]); EQ-5D-5L; and acceptability of the study design and treatment (assessed in an end-of-treatment questionnaire, focus groups, adverse event analysis, withdrawals, and compliance with allocated treatment).

Self-reported medication compliance was reported to the investigator at weeks 4, 12, and 24, aided by a participant-completed paper compliance diary. If participants missed more than 14 days in a month (cumulatively or consecutively), then this was considered clinically significant non-compliance (as this was felt to be a relevant threshold for this feasibility study).

All adverse events were recorded except for expected symptoms in the 48 h following influenza or SARS-CoV-2 vaccination as part of the NHS programme, and vaginal bleeding for those on active medication within the first 3 months of therapy or during the weaning period (these were recorded separately and categorised). An independent safety oversight clinician reviewed all safety events quarterly and reported to the Trial Management Group and Trial Steering Committee.

Criteria for progression to a fully powered, randomised, controlled trial were predefined as: (1) recruitment of at least 30 participants across all sites in 18 months (proportionately 22 participants were considered sufficient, owing to the COVID-19 pandemic, see Statistical analysis section); (2) a dropout rate of less than or equal to 30% of randomised individuals; and (3) acceptability to the majority of participants, including acceptable rates of adverse events.

At the onset of the COVID-19 pandemic, all study recruitment was paused for 5 months (March 16 to Aug 22, 2020). To enable re-opening, a protocol amendment (amendment 5) introduced an additional telephone screening call before the face-to-face screening visit (appendix p 2). Additionally, the baseline visit became remote (baseline outcomes were collected at the screening visit). Of note, most participants chose to receive either their first or second doses of a SARS-CoV-2 vaccine during the study period as these were offered to this age group at that time, as part of the UK's national vaccination programme.

### Statistical analysis

The study was not powered to test efficacy, but to test rates and feasibility of recruitment, randomisation, and retention. We estimated that recruitment of 60–90 participants would also enable us to obtain a good estimate of the variability of the anticipated efficacy outcome for a full trial (mean hand pain), which would be used to inform the sample size for such a trial.^30^ Therefore, a ceiling recruitment target of 90 and a minimum of 30 consenting individuals was set as sufficient to test the primary outcomes. Owing to the COVID-19 pandemic and an anticipated reduced recruitment period of 12–15 months rather than 18 months, a proportionately reduced minimum sample size of 22 was felt to still be sufficient to test the primary feasibility outcomes. This was agreed in principle with the trials unit, funder, and trial steering committee.

All analyses followed the statistical analysis plan as described in the protocol. Primary outcomes (recruitment, randomisation, medication compliance, and retention rates) were calculated as proportions with corresponding binomial exact 95% CIs. All patient-reported and clinical outcomes were analysed on an intention-to-treat basis. Treatment effects were calculated as the difference in the outcome between the two groups and were presented together with 95% CIs with all models adjusted for clinical subtype of painful hand joint, study site, and baseline values. Missing data were imputed by scoring guidelines and pre-defined rules as specified by the corresponding questionnaire. A multivariable linear regression model was fitted for outcomes available at baseline and week 24 only. Counts of painful joints were fitted with a negative binomial model. All models were adjusted for type of painful hand joint and site. Continuous outcomes available at week 12 and 24 were analysed by a linear mixed-effects model adjusting for treatment, type of painful hand joint, and baseline values as fixed effects and repeated measures within participants as random effects. The model includes robust SEs based on recruitment sites as clusters, and a time-by-treatment interaction with time as a categorical variable. The sample size for a full trial was estimated using the SD observed from data at week 24 by both methods of patient-reported mean hand pain for the previous 14 days (diary and recall) and a minimum, clinically important difference of 0·8 (or a 15% reduction in pain).^9^, ^29^, ^31^ The two methods for collecting hand pain data were compared by Bland-Altman plots. STATA IC, version 16·1, was used for all analyses. The study was registered with ISRCTN (ISRCTN12196200) and ClinicalTrials.gov (NCT04036929). CONSORT guidance for reporting a pilot or feasibility trial was followed (appendix pp 33–34).

### Focus groups

An evaluation of the end-of-treatment questionnaire and study protocol facilitated the development of a semi-structured interview guide by a trained qualitative researcher (CML). Purposive sampling was used to select and invite participants to one of two online focus groups (appendix pp 29–30). These were held and recorded in Microsoft Teams, transcribed by the qualitative researcher (CML) and analysed using a codebook approach to thematic analyses (appendix p 29).^32^ NVivo 12 software (QSR International) was used to organise data but not for analyses. Additional written informed consent was required to take part.

### Role of the funding source

The funder of the study had no role in study design, data collection, data analysis, data interpretation, writing of the report, or the decision to submit the paper for publication.

## Results

**Figure 1:**
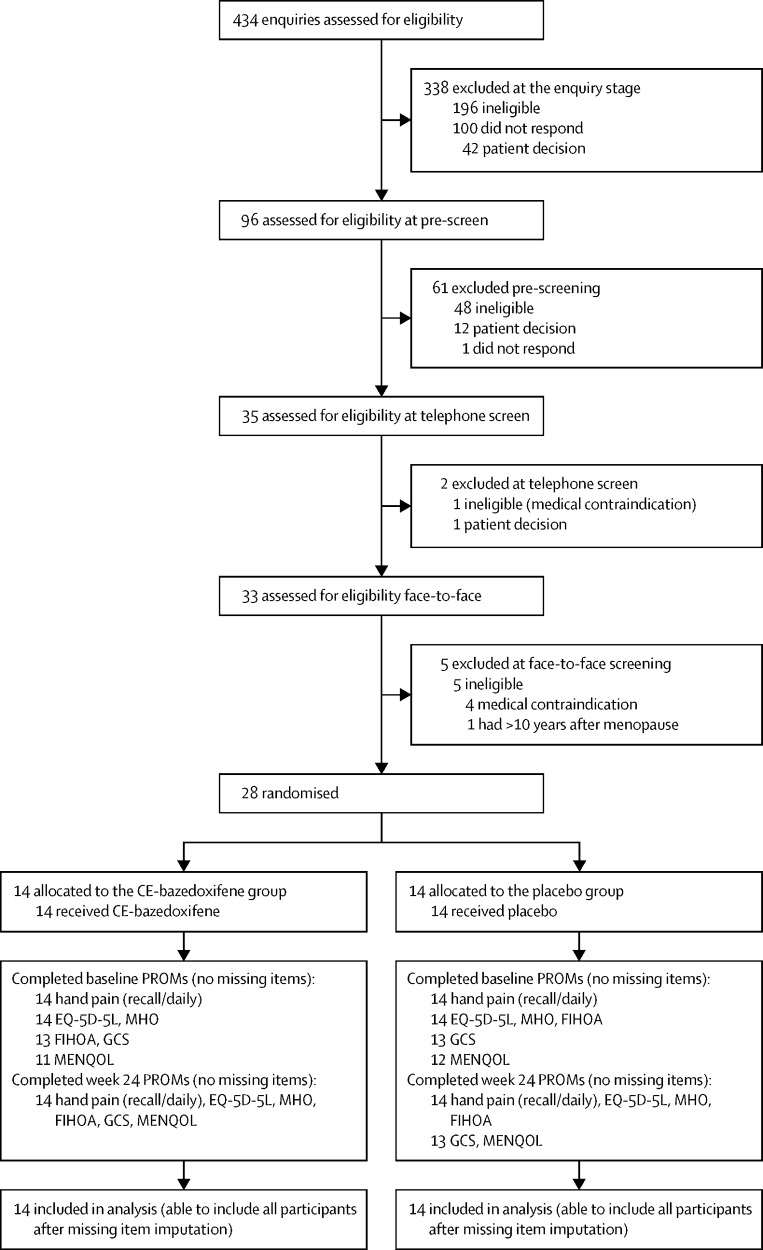
Trial profile CE-bazedoxifene=conjugated oestrogens plus bazedoxifene. PROM=patient-reported outcome measure. MHO=Michigan Hand Outcomes. FIHOA=Functional Index of Hand Osteoarthritis. GCS=Greene Climacteric Scale. MENQOL=Menopause-Specific Quality Of Life.

Between May 9, 2019 and Dec 31, 2020 (with a pause due to the COVID-19 pandemic from March 16 to Aug 22, 2020), we received 434 patient enquiries or clinician referrals (figure 1). Of these, 245 (56%) were ineligible, 101 (23%) did not respond, and 55 (13%) decided not to participate. Of the remaining 33 individuals who provided written informed consent and were assessed for eligibility face-to-face, five were ineligible and 28 were randomly assigned (figure 1). 14 participants were allocated to each intervention group with minimisation factors well balanced between groups (table 1). The last participant was randomly assigned on Feb 8, 2021, and follow-up continued until Aug 19, 2021. The mean age of the participants was 58·6 years (SD 3·4), with an average of 6·6 years since their final menstrual period. Most participants were white (27 [96%] of 28), were in full-time or part-time work (22 [79%]), and had painful hand osteoarthritis in both interphalangeal and base-of-thumb joints (19 [68%]). Those in the conjugated oestrogen plus bazedoxifene (CE-bazedoxifene) group had slightly higher mean hand pain at baseline than those in the placebo group (5·6 [1·3] *vs* 4·7 [1·1] on recall).

**Table 1:** Baseline characteristics and stratification factors of randomly assigned participants

		**CE-bazedoxifene (n=14)**	**Placebo (n=14)**
Age, years		58·9 (3·4)	58·3 (3·4)
Body-mass index, kg/m^2^		23·9 (3·7)	24·3 (3·0)
Average hand pain (recall)		5·6 (1·3)	4·7 (1·1)
Average hand pain (daily)		5·1 (0·9)	4·3 (0·9)
Duration of hand symptoms			
	<1 year	1 (7%)	4 (29%)
	1 year to <5 years	8 (57%)	6 (43%)
	5 years to <10 years	3 (21%)	0
	≥10 years	2 (14%)	4 (29%)
Type of painful hand joint*			
	Base of thumb only	1 (7%)	1 (7%)
	Interphalangeal joint only	4 (29%)	3 (21%)
	Interphalangeal joint plus base of thumb	9 (64%)	10 (71%)
Osteoarthritis elsewhere†			
	None	5 (36%)	3 (21%)
	Knee	3 (21%)	5 (36%)
	Hip	3 (21%)	3 (21%)
	Other (eg, elbow, shoulder, spine, or foot)	4 (29%)	6 (43%)
Ethnicity‡			
	White	13 (93%)	14 (100%)
	Other	1 (7%)	0
Employment status			
	Full time	8 (57%)	3 (21%)
	Part time	4 (29%)	7 (50%)
	Not working	2 (14%)	0
	Retired	0	2 (14%)
	Other	0	2 (14%)
Job activity (past or current)†			
	Heavy manual	2 (14%)	4 (29%)
	Repetitive use of hands	6 (43%)	5 (36%)
	Prolonged keyboarding	11 (79%)	8 (57%)
	None of these	1 (7%)	3 (21%)
Handedness			
	Left	0	1 (7%)
	Right	13 (93%)	13 (93%)
	Ambidextrous	1 (7%)	0
Smoking status			
	Never	10 (71%)	7 (50%)
	Current	2 (14%)	0
	Ex-smoker of ≤10 years	0	3 (21%)
	Ex-smoker of >10 years	2 (14%)	4 (29%)
First-degree family history of osteoarthritis†			
	None	5 (36%)	2 (14%)
	Hand	9 (64%)	10 (71%)
	Other (eg, knee or hip)	3 (21%)	5 (36%)
	Unknown	0	1 (7%)
Time from hysterectomy, years		9·2 (3·9–30·5;n=3)	NA
C-reactive protein, mg/L§		0·8 (0·2–1·2)	1·0 (0·6–2·7)
Follicle-stimulating hormone, IU/L		71·0 (64·1–78·4)	74·4 (58·3–104·4)
Medical history¶			
	Hypertension	2 (14%)	2 (14%)
	Vascular‖	0	1 (7%)
	Diabetes	0	1 (7%)
	Hyperlipidaemia	2 (14%)	3 (21%)
	Renal or liver disease	3 (21%)	0
	Basal cell carcinoma, fully treated	2 (14%)	0
Concomitant medications			
	Topical NSAID	3 (21%)	7 (50%)
	Paracetamol	6 (43%)	5 (36%)
	Oral NSAID	4 (29%)	6 (43%)
	Co-codamol	1 (7%)	1 (7%)
	Intra-articular steroid	1 (7%)	0
	Opioids	0	0
	Other	6 (43%)	8 (57%)
	Participants with one or more concomitant medications	9 (64%)	13 (93%)
Centre*			
	Nuffield Orthopaedic Centre	7 (50%)	7 (50%)
	White Horse Medical Practice	5 (36%)	4 (29%)
	Charing Cross Hospital	2 (14%)	3 (21%)

Data are n (%), mean (SD), or median (IQR). CE-bazedoxifene=conjugated oestrogens plus bazedoxifene. NA=not applicable. NSAID=non-steroidal anti-inflammatory drug.

* Stratification factor.

† Participants could select more than one category.

‡ Self-reported ethnicity; ethnicities could be selected as white, southeast Asian, south Asian, Afro-Caribbean, and other (only white and other were selected).

§ One participant had C-reactive protein <0·2 mg/L.

¶ Where these medical conditions were present, they were controlled or treated.

‖ One or more of stroke or angina, venous thromboembolism, or cardiovascular disease.

Of the 434 patient enquiries and clinician referrals received, 375 (86%) were self-referred patient enquiries from the community and 59 (14%) were clinician referrals. The recruitment sources with the highest proportions of random assignments were the online local newspaper (two [18%] of 11), word of mouth (four [16%] of 25), and websites (seven [11%] of 62), whereas only four (7%) of 59 clinician referrals resulted in a participant being randomly assigned (appendix p 9). The recruitment sources with the highest absolute number of random assignments were websites (7) and text messages following general practitioner (GP) database search (5). 28 (6%) of 434 enquiries or referrals resulted in random assignment. The proportion of random assignments at the primary care site (9 [32%] of 28) was lower than that at the lead secondary care site (14 [50%]) but greater than that at the other secondary care site (5 [18%]). The time open to recruitment at different sites is shown in the appendix (p 10).

There was an increase in recruitment rate (proportion randomly assigned from those screened) following amendments made due to low recruitment and the COVID-19 pandemic (appendix p 11). Of the 35 patients who completed any type of screening, eight were screened before the amendments were made, with five randomly assigned, giving a recruitment rate of 63% (95% CI 24–91). After the amendments were made, 27 patients were screened and 23 were randomly assigned, giving a recruitment rate of 85% (66–96). Overall, the recruitment rate was 80% (63–92). There was an average of two participants randomly assigned per month over 15 months (appendix p 10). There was a 100% completion rate (100–100) in both methods of hand pain measurement (appendix p 12). The daily mean hand pain method had high completion rates (median number of scores 14 [IQR 13–14]), with no participants recording less than 12 of 14 possible ratings at either baseline or week 24 (appendix p 12).

406 enquiries or referrals did not result in the individual being randomly assigned to a treatment group. 250 (62%) of 406 patients were ineligible to take part, with contraindicated medications accounting for 50 (20%) of these 250 (appendix p 13). 55 (14%) of 406 patients decided not to take part, with the most common reason being not wanting to take a hormone-based drug. In addition, 101 (25%) of 406 stopped communicating with the study team after initial enquiry (figure 1, table 2).

**Table 2:** Feasibility of recruitment and reasons for exclusion from study

		**Enquiry**	**Pre-screen**	**Telephone screen**	**Face-to-face screen**	**Total**
Total participants excluded		338	61	2	5	406
Ineligible*		196 (58%)	48 (79%)	1 (50%)	5 (100%)	250 (62%)
	Contraindicated medication†	47 (24%)	3 (6%)	0	0	50 (20%)
	Medical contraindication‡	26 (13%)	10 (21%)	1 (100%)	4 (80%)	41 (16%)
	>10 years after final menstrual period	23 (12%)	9 (19%)	0	1 (20%)	33 (13%)
	Aged >65 years	27 (14%)	0	0	0	27 (11%)
	<12 months after final menstrual period	23 (12%)	4 (8%)	0	0	27 (11%)
	Hand pain reported <4/10	13 (7%)	8 (17%)	0	0	21 (8%)
	Other cause of hand pain	14 (7%)	6 (13%)	0	0	20 (8%)
	Only one joint affected	7 (4%)	1 (2%)	0	0	8 (3%)
	No formal clinical diagnosis of osteoarthritis	3 (2%)	4 (8%)	0	0	7 (3%)
	Other	4 (2%)	1 (2%)	0	0	5 (2%)
	NICE core management guidance not tried	3 (2%)	2 (4%)	0	0	5 (2%)
	Aged <40 years	2 (1%)	0	0	0	2 (1%)
	Unknown	4 (2%)	0	0	0	4 (2%)
Participant decision		42 (12%)	12 (20%)	1 (50%)	0	55 (14%)
	Did not want to take a hormone-based drug	10 (24%)	4 (33%)	0	0	14 (25%)
	Difficulty attending study visits	11 (26%)	1 (8%)	0	0	12 (22%)
	Personal reasons	3 (7%)	4 (33%)	0	0	7 (13%)
	Unknown	5 (12%)	0	1 (100%)	0	6 (11%)
	General concern about taking drugs	4 (10%)	0	0	0	4 (7%)
	Insufficient time to participate	3 (7%)	0	0	0	3 (5%)
	Did not want to risk taking placebo	3 (7%)	0	0	0	3 (5%)
	Other	3 (7%)	3 (25%)	0	0	6 (11%)
No response		100 (30%)	1 (2%)	0	0	101 (25%)

Data are n or n (%).

* This is the primary reason for ineligibility, listed according to the first reached in the order of inclusion and exclusion criteria in the protocol (appendix pp 5–6); some people had multiple reasons.

† Contraindicated medications were recorded as: existing hormone replacement therapy (n=42), systemic hormonal contraceptive (n=3), epilepsy medication (n=3), selective oestrogen receptor modulator (n=1), and steroid injection (n=1; appendix p 13); people could have more than one contraindicated medication, which are shown according to the first reached in the exclusion criteria (appendix pp 5–6).

‡ Primary medical contraindications were recorded as: migraine (n=12), history of breast, endometrial, ovarian, or skin cancer (n=9), body-mass index more than 30 kg/m^2^ (n=9), active or past history of arterial thromboembolic disease or strong family history of stroke (n=4), history of other cancer within 5 years (n=3), uncontrolled hypertension (n=2), uncontrolled hypertriglyceridaemia (n=1), and acute liver disease (n=1; appendix p 13).

By protocol definition, there was 100% self-reported compliance with the medication in the CE-bazedoxifene group (14 of 14) and 93% compliance in the placebo group (13 of 14), due to one participant's withdrawal from treatment before the 12-week follow-up. There were three withdrawals from treatment during the study, all in the placebo group (appendix p 14). The remaining two withdrawals occurred at the time of weaning of the study medication (24-week visit). There were nine protocol deviations across eight participants, balanced across treatment groups (appendix p 15).

In the placebo group, 50% of participants could correctly guess their treatment at the end of the study, which is equivalent to chance (participant Bang's blinding index 0·50 [95% CI 0·25–0·75]; appendix p 16). There was no evidence of participants guessing their treatment beyond chance in the CE-bazedoxifene group and no evidence that the investigator was able to guess the participant's treatment beyond chance in either group. There were two incidences of intentional unmasking for safety, both of which were in the placebo group, and two incidences of accidental unmasking (appendix p 17).

**Figure 2:**
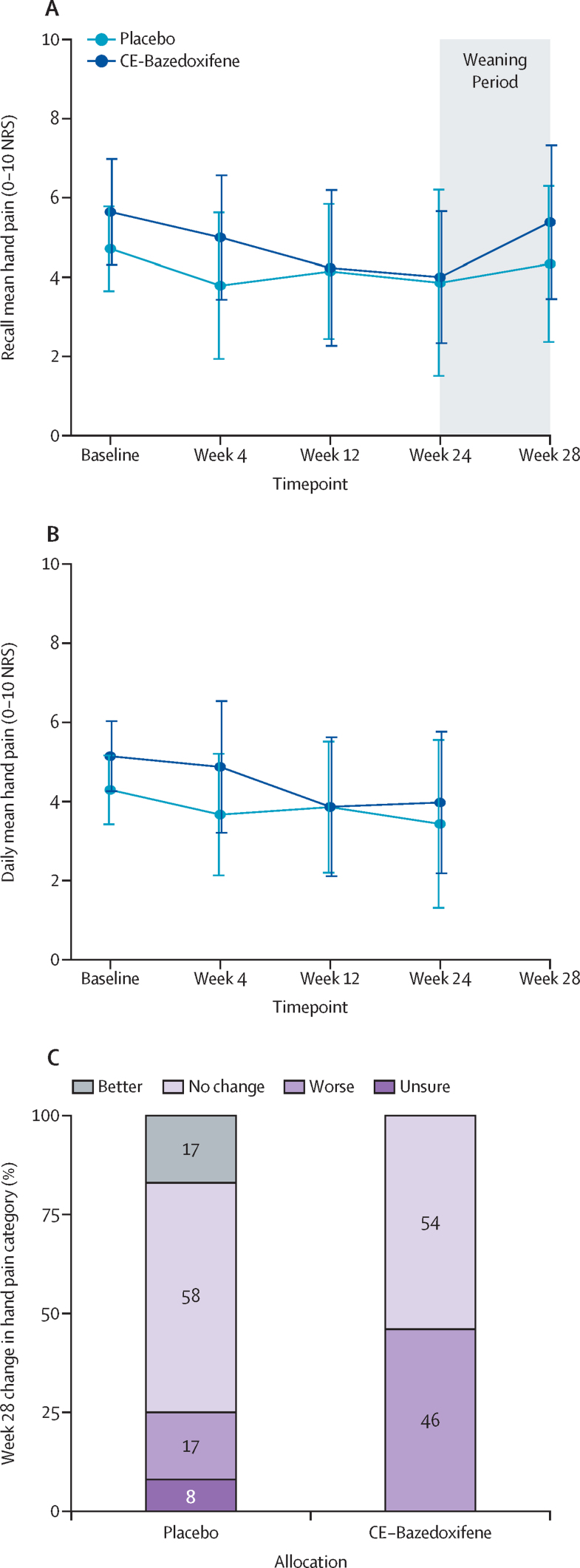
Change in mean hand pain in participants over the study period (A) Recall at study visits of mean hand pain on a 0–10 NRS for the preceding 14 days before the baseline, and week 4, 12, 24, and 28 study visits. (B) Mean of recorded daily mean hand pain on a 0–10 NRS for the preceding 14 days before the baseline, and week 4, 12, and 24 study visits (not collected at week 28). The point is the mean and error bars represent the SD. (C) Patient-reported category of change in hand pain for the preceding 14 days before the week 28 study visit (following weaning from study medication), by treatment allocation. The proportion within the category is shown on bars. CE-bazedoxifene=conjugated oestrogens plus bazedoxifene. NRS=numerical rating scale.

**Figure 3:**
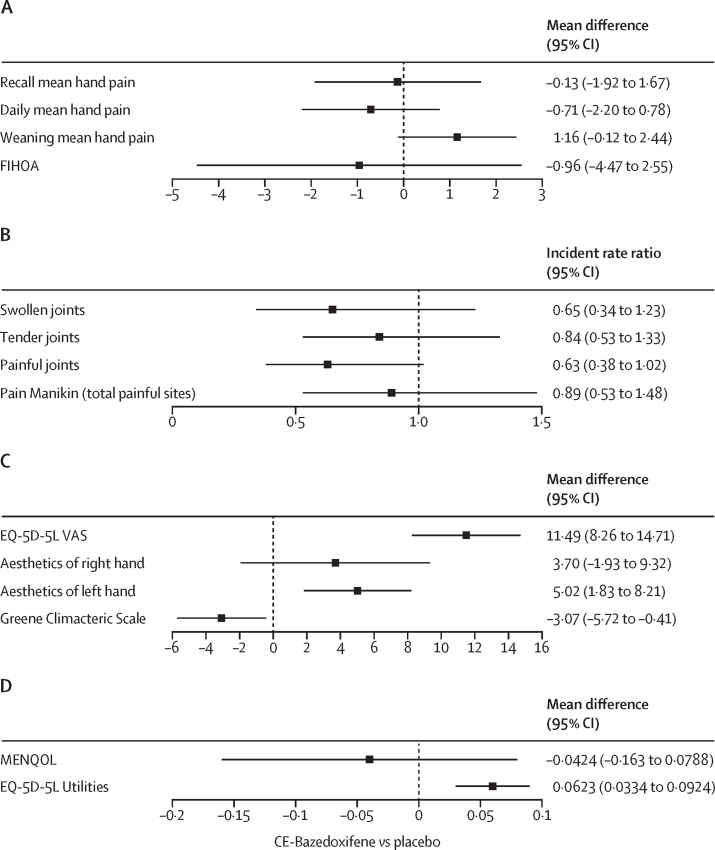
Treatment effects on secondary outcomes in study participants For all outcomes, the mean difference between baseline and week 24 is shown unless otherwise stated, considering the treatment effect of CE-bazedoxifene versus placebo. Panels are grouped by scale of outcome. (A) Hand pain outcomes. Recall and daily mean hand pain are shown; weaning mean hand pain is the difference between week 28 and week 24 recall mean hand pain (all measured on a 0–10 NRS, so lower scores indicate less pain). FIHOA (range from 0–30, higher scores indicate worse performance). (B) Joint outcomes; lower scores indicate lower joint counts (range 0–32) or fewer painful sites on pain manikin (range 0–14). (C) EQ-5D-5L VAS range is 0–100, higher scores indicate improved quality of life. GCS range is 0–63 (lower scores indicate less bothersome symptoms). Aesthetics of right and left hand were taken from cosmesis questions of the Michigan Hand Outcome questionnaire (range 0–100, higher scores indicate better hand appearance). (D) MENQOL average of domains shown, range 1–8 (lower scores indicate less bothersome symptoms). EQ-5D-5L scores range from –0·594 to 1 (higher scores indicate better health). CE-bazedoxifene=conjugated oestrogens plus bazedoxifene. FIHOA=Functional Index of Hand Osteoarthritis. VAS=Visual Analogue Scale. NRS=Numerical Rating Scale. GCS=Greene Climacteric Scale. MENQOL=Menopause-Specific Quality of Life.

There was a high completion rate for the patient-reported outcomes (appendix p 18). By both methods, mean hand pain decreased from baseline to week 24 in both treatment groups (figure 2A–B), and these methods showed good agreement (appendix p 19). The two methods agreed that there was no significant evidence of a treatment effect, noting that the study was a feasibility study and thus not powered to detect such an effect (change in recall mean hand pain –0·13 [95% CI –1·92 to 1·67]; daily mean hand pain –0·71 [–2·20 to 0·78; figure 3A]).

The study was not powered to detect a treatment difference, but we report results from the secondary outcomes as an indication of treatment effect and to help with planning of the full study. The recall mean hand pain increased by 1·31 points in the CE-bazedoxifene group from week 24 to week 28 (during the period when participants weaned and stopped the medication) compared with 0·17 points in the placebo group (figure 3A), indicating a possible effect of cessation of medication. This observation was supported by the proportions of participants reporting a change in hand pain after weaning: six (46%) of 13 participants in the CE-bazedoxifene group felt their pain was worse at week 28 compared with week 24, but only two (17%) of 12 participants reported worsening in the placebo group (figure 2C). Results for all other secondary outcomes—ie, quality of life by EQ-5D-FL utilities; hand function by FIHOA; grip strength; counts of painful, swollen, and tender joints; total number of painful body sites; hand aesthetics by MHO; menopause symptoms by GCS and MENQOL; and global impression of change—are shown in figure 3C–D and the appendix (pp 20–26).

Although they were not included based on symptoms of menopause, it is of note that the study population as a whole had evidence of troublesome menopausal symptoms at baseline, seen on both measures (GCS and MENQOL; figure 3C–D; appendix p 25). As might be expected due to its licensed indication, there was some evidence that CE-bazedoxifene reduced overall symptoms in the total GCS score (figure 3C) and some subdomains on both menopause symptom measures (appendix p 25), but this might have occurred by chance.

Satisfaction and dissatisfaction with the medication according to the patient-reported questionnaire at week 24 was similar in both treatment groups (appendix p 27). There were overall good or high levels of satisfaction with taking part in the study. 26 (93%) of 28 would recommend taking part to others with hand osteoarthritis (irrespective of treatment group; appendix p 28). Many found the flexibility offered by a combination of remote and face-to-face visits (introduced due to the COVID-19 pandemic) attractive.

Two online focus groups (ten participants in total: four in group 1 and six in group 2) were held. From 45 early codes from thematic analysis of the transcripts, three main categories and 16 areas of recommendation for refinement for a full trial were developed (appendix pp 29–32). These areas of recommendations included considering hand stiffness as well as pain measures, considering a single-hand rating of pain (rather than an average of both hands) on a daily basis, recognising taking photographs of their hands could be upsetting or uncomfortable for participants, reducing the overall number of questionnaires or questions, standardising and including hand exercises and other pieces of advice at the start of the study, and considering a longer follow-up period after the intervention.

There were 69 adverse events recorded in the study across 25 (89%) of 28 participants (13 in the placebo group and 12 in the CE-bazedoxifene group). Of these, 13 were judged to be treatment-related (ahead of unblinding) and affected nine participants (subsequently shown to be five in the placebo group and four in the CE-bazedoxifene group; table 3). There were no serious adverse events recorded during the study.

**Table 3:** Summary of adverse events and bleeding events

		**CE-bazedoxifene (n=14)**	**Placebo (n=14)**	**Total (n=28)**
**Related adverse events**				
Number of participants with treatment-related adverse event		4 (29%)	5 (36%)	9 (32%)
Total number of treatment-related adverse events		6	7	13
	Breast*	2 (33%)	0	2 (15%)
	Gastrointestinal	0	1 (14%)	1 (8%)
	Metabolism	1 (17%)	1 (14%)	2 (15%)
	Musculoskeletal	0	1 (14%)	1 (8%)
	Neurological	1 (17%)	1 (14%)	2 (15%)
	Reproductive system	0	1 (14%)	1 (8%)
	Skin	1 (17%)	1 (14%)	2 (15%)
	Vascular	1 (17%)	1 (14%)	2 (15%)
**Unrelated adverse events**				
Number of participants with unrelated adverse event		12 (86%)	13 (93%)	25 (89%)
Number of adverse events unrelated to study treatment		26	30	56
	Cardiovascular	1 (4%)	0	1 (2%)
	Dental	1 (4%)	0	1 (2%)
	Gastrointestinal	1 (4%)	0	1 (2%)
	Metabolism	1 (4%)	1 (3%)	2 (4%)
	Musculoskeletal	9 (35%)	4 (13%)	13 (23%)
	Neurological	6 (23%)	7 (23%)	13 (23%)
	Ophthalmology	0	1 (3%)	1 (2%)
	Psychiatric	0	3 (10%)	3 (5%)
	Reproductive system	0	3 (10%)	3 (5%)
	Respiratory	2 (8%)	5 (17%)	7 (13%)
	Skin	4 (15%)	1 (3%)	5 (9%)
	Urological	0	2 (7%)	2 (4%)
	Vascular	1 (4%)	3 (10%)	4 (7%)
**Episodes of bleeding**				
Number of episodes of bleeding		0	3	3
Number of participants with episodes of bleeding		0	2 (14%)	2 (7%)
Number of days of bleeding†		..	1 (0)	1 (0)

Data shown are n (%) or mean (SD). Related adverse events were judged to be possibly, probably, or definitely related to treatment by a clinical investigator. Unrelated adverse events were judged to be unlikely to be or definitely not related to treatment. Relatedness was judged ahead of unmasking in all cases. CE-bazedoxifene=conjugated oestrogens plus bazedoxifene.

* Adverse events related to the breast were mild breast tenderness not requiring participants to stop study medication in both cases.

† One participant had ongoing bleeding at the 28-week follow-up; this was followed to resolution.

When considering the criteria for progression to a full study, all three were met, as reviewed by the Trial Steering Committee on Feb 8, 2022. The sample size for a full trial, based on a minimum clinically important difference of 0·8 points on the pain numerical rating scale, was estimated as 296, assuming an SD of 2, 90% power, and 10% loss to follow-up.^9^, ^29^, ^31^

## Discussion

Our study provides evidence for the first time that a trial of the effects of an oral HRT on musculoskeletal symptoms in a population of post-menopausal women with painful hand osteoarthritis is both feasible and acceptable. Our study met its pre-defined progression criteria. Recruitment was improved by a number of protocol amendments, which might help other studies in this area: notably the broadening of inclusion criteria to those with only base-of-thumb osteoarthritis (although still a minority of study participants), increasing methods for recruitment in the community (particularly use of text messages from GP surgeries and website presence), and increased remote activity brought about by the COVID-19 pandemic. The option for remote study visits was popular and, although it led to some missing secondary outcomes at the 4-week and 12-week visits, this did not compromise safety monitoring or planned analyses. Remote study visits might have enabled more working people or those with other commitments to take part, something that is important for clinical trials to ensure generalisability and align with the National Institute for Health Research INCLUDE agenda. Large numbers of people needed to be identified to randomly assign 28 individuals, given a 6% conversion rate from identification to random assignment. However, this ratio (1:10 to 1:20 of those identified) is what is seen in other UK interventional osteoarthritis studies.^33^ This conversion rate should be kept in mind when designing and considering recruitment strategies in osteoarthritis trials. Recruitment to osteoarthritis studies often seems disproportionately challenging for one of the most common musculoskeletal conditions. As evident from our study population, many people living with the condition are not in secondary or even primary care, so they need to be identified via other routes, such as general community advertising.

What insight can be gained from the secondary outcome data in this study? It must be reiterated that this study was not powered to test or demonstrate efficacy of this agent on any of the outcomes collected. Our ceiling target for recruitment would have increased power to do this, but numbers were below this target, in part due to the pandemic. Although women were not included based on their menopausal symptoms (hence why drug use was outside of license), given our inclusion of women between 1 and 10 years after menopause, many had considerable menopausal symptoms at baseline. An improvement in EQ-5D-5L (quality of life) and various domains on MENQOL and overall score on the GCS were seen. The selected agent would be expected to have these effects given its license, potentially demonstrating our sensitivity to detect change (although this could have occurred by chance).^21^ How relevant the presence of other features of the menopausal syndrome were to the presence or response of musculoskeletal outcomes is an important question going forwards (and which was not possible to assess in this study).

A full trial would require substantial investment. A future study might wish to extend the treatment period to longer than 6 months to maximise detection of any treatment effect. It would be wise to study the period of stopping and include longer follow-up off the drug to examine the occurrence of any flares on cessation. In a recent UK study using electronic health-care data, increased incidence of hand osteoarthritis was seen within the 18 months following HRT cessation, perhaps supporting this phenomenon.^15^ Our participants were weaned over a 4-week period; a longer period might be preferable, particularly if the treatment period was extended. Furthermore, proposed refinements and changes for a number of areas were identified by the qualitative work at the end of the study. It would be our recommendation that these are carefully considered and incorporated wherever possible into the design of a randomised controlled trial.

This study was not designed to test the potential mechanism of the effects of oestrogens or selective oestrogen receptor modulators in this setting, but it would seem important to consider these in the design of a future trial. Hand pain was chosen as an indicative primary outcome as it is important to patients. Beyond the evidence for oestrogen insufficiency being temporally associated with the development of hand osteoarthritis, there is evidence that replacement of oestrogens can improve musculoskeletal pain. In large HRT trials (Women's Health Initiative and an Australian randomised controlled trial), all-cause musculoskeletal pain improved in the oestrogen-only group (although it is not possible to know how much of this was osteoarthritis or hand osteoarthritis).^14^, ^34^ Those on oestrogen only had reduced incidence of musculoskeletal symptoms.^35^ Oestrogens and other sex hormones are known to modulate all levels of pain pathways (descending pathways, dorsal horn, and peripheral pain-sensing all being targets).^36^ Pain (including musculoskeletal pain) is known to be modulated by a variety of factors, including sleep, stress, and anxiety. The proven effects of HRT on such factors might also be important in the context of both musculoskeletal pain and wellbeing outcomes.^37^ Furthermore, oestrogens tend to induce anti-inflammatory cytokines and immunomodulatory pathways. Such anti-inflammatory effects might be relevant to those with joint pain.^38^ It might be that the measurement of hormonal profiles or systemic inflammation in individuals might allow further stratification as part of a future trial. We did not seek to test effects on joint structure or tissues specifically (although measures of hand pain, function, or swelling might all indirectly do this). The known anabolic effects via oestrogen receptors of oestrogens or selective oestrogen receptor modulators on connective tissues, including articular cartilage and bone, and the reported protective effect of oestrogens on progression to arthroplasty in the Women's Health Initiative would suggest that a structural modification by this drug class could be possible.^13^, ^17^ However, detection of such a structural effect using outcomes approved by the US Food and Drug Administration (ie, X-ray) would require a different study design and far greater numbers than were included in this study, making it less feasible.

In terms of the best way to measure patient-reported outcomes, daily rating of hand pain was acceptable to patients (moreover, the patient and public involvement discussion group requested it) and provided high rates of data completeness. It correlated closely with recalled hand pain in our study (although associations are biased as these measures were not collected independently). Daily hand pain rating gave smaller measures of variation, suggesting that this measure might be more reliable and more sensitive to change.^29^ However, rating hand pain was not meaningful for all patients, and those with disparities between the two hands would have preferred separate ratings for each hand (from which an average could be derived). For some, the symptom of hand stiffness rather than pain would be more understandable, and a patient preference measure could be considered.

Our study had some limitations. Aside from the number of participants being at the lower end of the target, limiting what can be gleaned from proof-of-concept data, the generalisability of our findings is difficult to judge. The acceptability measures (by qualitative work and end-of-study questionnaire) were obtained from a small percentage of people who took part in the study, probably leading to increased approval rates. Qualitative work in those who were eligible to take part but chose not to would have been insightful. The low number of participants on active medication (n=14) meant that some safety events might not have occurred by chance (for example, we would have expected some cases of vaginal spotting or bleeding). Our recruitment spanned the COVID-19 pandemic; although the pandemic negatively impacted recruitment (for example, due to site closures or hesitancy to attend hospital sites), it is also clear that the world became more aware of clinical trials and their importance in identifying new treatments, because of the high profile of vaccine trials. This awareness could have positively benefited clinical trial participation in some sectors. It is difficult to know if this effect will last. People were often working from home, sometimes with more time than usual, with increased health vigilance leading to increased chances of participation. Due to the pandemic, there might have been biases introduced, such as the ability to see a medical investigator more often as part of their participation. Therefore, it is difficult to extrapolate these study data fully to a larger trial beyond the pandemic. The majority of participants received at least one dose of a SARS-CoV-2 vaccine during their participation, although none in the immediate period before their baseline or 24-week visits. Lastly, the delivery of this study was done by individuals with a high interest and expertise in both musculoskeletal and women's health, which was seen as important to participants. Trials that span clinical areas that are usually disparate, such as these, are challenging, and site team training will be essential in successfully scaling up. Interdisciplinary working has the potential for very real patient benefit, providing opportunities to understand disease and develop novel therapeutic options. Beyond academia, research, development, and policy challenges remain—in government, in the pharmaceutical industry, and in society—to consider these two areas of musculoskeletal health and women's health together. Whether in electronic health records, large cohorts, or clinical trials, collecting and coding high-quality musculoskeletal and menopause data is critical to improve our knowledge going forward.

In summary, these results provide justification for progression to a full efficacy trial along with considerations for its refinement. Due to the high cost of clinical trial drug manufacture of this class of agents (sex hormones have manufacturing restrictions), it is likely to be essential that there is pharmaceutical industry involvement in any future full trial. Successful recruitment to such studies requires increased public awareness of this condition and of musculoskeletal clinical trials, with better national mechanisms for highlighting participation opportunities to the community living with osteoarthritis than currently exist. An additional consideration is how an efficacious agent would be implemented into health-care systems where there are currently shortages of some HRTs and restrictions on the number and nature of these agents made available to women. There remains a high unmet need in osteoarthritis and other common musculoskeletal conditions to find new treatments to prevent or slow conditions and to help relieve pain.^39^ Beyond this, there is an unmet need to better understand sex-specific differences in musculoskeletal conditions and whether there are identifiable subgroups within conditions who would benefit from more tailored treatments.

## Data sharing

Access to the de-identified dataset for purposes of research other than this study would be at the discretion of the chief investigator (FEW) and OCTRU. All participants have consented to the information collected about them from the study being used in a de-identified form to support other research on hand osteoarthritis in the future and which might in some circumstances be passed on to other collaborators of the research team in organisations other than the University of Oxford, which may include those outside the EU and commercial organisations. Requests for the de-identified dataset generated during the current study should be made to the chief investigator (FEW) or OCTRU, at octrutrialshub@ndorms.ox.ac.uk. FEW and OCTRU will consider requests once the main results from the study have been published up until Dec 10, 2036. All requests must relate to bone fide research into hand osteoarthritis research.

## Declaration of interests

MVG is supported in part by a scholarship from the Clarendon Fund (Oxford University Press). TLV has received research grant support from Pfizer, Biosplice, Galapagos, Fidia, UCB, and Novartis. FEW received personal consulting fees in 2020 from Pfizer for an unrelated compound; her institution received funding from Versus Arthritis for her role as lead of a Versus Arthritis Research Advisory Group. KV has received research grant support from Bayer Healthcare, consulting fees from Bayer Healthcare, Grunenthal GmBH, and AbbVie, and honoraria or speaker fees from Bayer Healthcare, Eli Lilly, and Grunenthal GmBH. All other authors declare no competing interests. Pfizer did not support, directly or indirectly, any part of the study.
