# Supplementary material for: Hormone replacement therapy (conjugated oestrogens plus bazedoxifene) for post-menopausal women with symptomatic hand osteoarthritis: primary report from the HOPE-e randomised, placebo-controlled, feasibility study
Source: Lancet Rheumatol. 2022 Sep 21;4(10):e725–37. doi: 10.1016/S2665-9913(22)00218-1 (PMC9620575; doi:10.1016/S2665-9913(22)00218-1)
Supplement: Supplementary appendix [file mmc1.pdf]

THE LANCET
Rheumatology

Supplementary appendix

This appendix formed part of the original submission and has been peer reviewed. We post it as supplied by the authors.

Supplement to: Williams JAE, Chester-Jones M, Lowe CM, et al. Hormone replacement therapy (conjugated oestrogens plus bazedoxifene) for post-menopausal women with symptomatic hand osteoarthritis: primary report from the HOPE-e randomised, placebo-controlled, feasibility study. *Lancet Rheumatol* 2022; **4:** e725–37.

Supplementary Appendix

Contents

Supplementary Table 1. Protocol amendment history for the study……………………………………………..….........2

Supplementary Table 2. Full eligibility criteria for the study……………………………………………….....................5

Supplementary Figure 1. Study visit schedule flow chart…………………………………………….........................7

Supplementary Table 3. Rates of recruitment from different sources……………………………………….……………...9

Supplementary Table 4. Randomisations per month, by site……………………………….………….................10

Supplementary Table 5. Recruitment rates pre/post amendments 4 and 5…………………………………….….…..11

Supplementary Table 6. Retention rates, based on collection of mean hand pain…………………………………………12

Supplementary Figure 2. Reasons for ineligibility (medication and medical contraindications) …………………………13

Supplementary Table 7. Withdrawals from treatment during the study…………………………………….………..14

Supplementary Table 8. Protocol deviations during the study……………………………….………….............15

Supplementary Table 9. Participant and Investigator response to blinding questions at week 24 and corresponding Bang's Blinding Index…………………………….…………...16

Supplementary Table 10. Accidental and intentional unblinding………………………….……………........17

Supplementary Table 11. Completeness of patient reported outcome measures at Baseline and Week 24……………….18

Supplementary Figure 3. Comparison of two methods of rating mean hand pain during the study…………………...….19

Supplementary Table 12. Health quality of life and hand function in participants over time……………………………..20

Supplementary Table 13. Grip strength in participants over time…………………………………………………………21

Supplementary Table 14. Tender, painful and swollen joints over time………………………………………………..22

Supplementary Table 15. Pain and function outcomes in participants over time…………………………………………23

Supplementary Table 16. Joint appearance measured by Michigan Hand Outcome questionnaire (aesthetic/cosmesis questions only) in participants over time……………………………………………………………………………..24

Supplementary Table 17. Menopause symptoms measured by MENQOL and Greene Climacteric Scale in participants over time……...25

Supplementary Figure 4. Patient reported Global impression of change at week 24 visit, by treatment arm……..……….26

Supplementary Figure 5. Satisfaction with medication responses, by treatment arm……………………………………..27

Supplementary Figure 6. End of study experience questionnaire…………………………………………………………28

Supplementary methods: Focus Groups ……….……………………………………………………………………29

Supplementary Table 18. Sampling frame defining the variables of the participants who participated in the two focus groups……………………………………………………………………………………………………...….30

Supplementary results: Focus Groups results...………………………………………………………………………31

Supplementary Table 19. Suggestions for consideration to inform a main trial from the focus group analysis…………..32

Supplementary Table 20. CONSORT, Feasibility extension checklist……………………………………………………33

References…….35

Amendment No.	Protocol Version No.	Date issued	Details of Changes made, including reason for change
8	8·0	08Nov2021	The Chief Investigator, Fiona Watt's title and job position have been updated in line with her honorary position within the department.<br>The Sponsor name has been updated following an internal team restructuring.<br>Catherine Minns Lowe has been added as an Investigator.<br>Section 8.8 (Subsequent Visits/Follow-up) The Focus Groups section has been updated:<br>• Participants will be sent an invitation letter and consent form if selected to take part in a focus group.<br>• Participants will be selected for the focus groups using purposive sampling by a qualitative researcher.<br>• Two researchers will be present at each focus group.<br>• Focus groups will be held online only and will be video as well as audio recorded.<br>• Questions that will be asked during focus groups will be developed following the analysis of the End of Treatment questionnaire.<br>• The qualitative researcher will write up the transcript.<br>Section 11.5 (Analysis of Outcome Measures) has been updated to include thematic analysis of the qualitative data.<br>Section 12.3 (Data Recording and Record Keeping) has been updated so that it mentions the video recording of the focus groups and that audio and video recordings will be deleted once transcribed.<br>Section 16 (Publication policy) has been updated because participants will be informed of their treatment allocation following final data lock and ahead of participating in a focus group.
7	7·0	12May2021	Section 10.2.1 (Adverse events – Specific Considerations) has been updated to include expected side effects within 48 h of a flu/COVID-19 vaccine do not need to be reported as adverse events.<br>Section 13.1.2 (Safety Oversight Clinician (SOC)) – removed the ability of the SOC to stop the study due to clear evidence of the effectiveness of a treatment.<br>Correction of typographical errors.
6	6·0	03Dec2020	Section 12.1 (Source Data), 12.3 (Data Recording & Record Keeping) and 14.5 (Participant Confidentiality) have been updated to include the pre-screening proforma.<br><br>Removal of Pulvertaft Hand Centre (University Hospitals of Derby and Burton NHS Foundation Trust) as a fourth research site
5	5·0	20Jul 2020	Section 6.1 added summarising the COVID-19 related changes that have been made to the study visits to minimise planned face-to-face visits.<br><br>The Screening Visit has been split into two parts and is now referred to as the Screening Assessment: a Screening Telephone Call, and a Screening Face-to-Face Visit.<br>The Screening Assessment visit window has been extended so that the Screening Telephone Call can be performed -70 days prior to the Baseline Visit.<br><br>Clearly stated that Screening X-rays are optional and are only performed if participant agrees.<br><br>Baseline outcomes will be collected at the end of the Screening Face-to-Face Visit.<br><br>Section 8.7.1 added detailing that the Baseline, Week 4, Week 12, and Week 24 Visits have the option to be carried out remotely via telephone.<br><br>Participants will receive an additional short phone call prior to each study visit to assess and document whether a remote telephone call or face-to-face visit is recommended.<br><br>Participant questionnaire packs and study paper diaries will be sent out to participants via post where a remote telephone call is scheduled for a study visit.<br>These will be returned in pre-paid envelopes.<br><br>Added the option for participants to collect their 2 prescriptions of study medication directly from the site (without needing to attend a Baseline Visit or Week 12 Visit).<br><br>Some procedures (physical examination, safety blood monitoring, urine dipstick, urine pregnancy testing, Investigator-recorded painful, tender, and swollen joint counts, photographic recording of hands, Jamar grip strength) will not be performed at study visits where the study visit is carried out remotely via telephone.<br><br>Section 8.8.3 added detailing the changes to sample collection due to COVID-19.<br><br>Option added for remote collection of blood pressure and photographic recording of hands where the study visit is performed remotely.<br><br>Option added that focus groups may be conducted remotely using a web-based platform.<br><br>Focus group discussion themes updated to include the acceptability of remote telephone calls

			versus face-to-face visits.<br><br>Added to Section 8.10 that a participant may be withdrawn from treatment due to considerations relating to the COVID-19 pandemic.<br><br>Section 10.2.1 updated to reflect that the ability to respond to and investigate post—menopausal bleeding will initially be by telephone.<br><br>Section 11.5.1 added to reflect the changes to data collection and analysis due to the COVID-19 changes.<br><br>Schedule of Procedures 2 - Modified Screening Assessment and Remote Study Visits added to Appendix B.<br><br>Appendix D added detailing the considerations for the decision-making process for a remote telephone or a face-to-face study visit.
4	4·0	30Apr 2020	Updates to Investigator list<br>Addition of Chelsea & Westminster Hospital as a secondary care Participant Identification Centre<br>Addition of Pulvertaft Hand Centre (University Hospitals of Derby and Burton NHS Foundation Trust) as a fourth research site<br>Removal of reference to specific sites in regards to ability to carry out out-of-hours unblinding via trials pharmacy
3	3·0	06Dec 2019	Clarification to inclusion criteria (7.2) that the criteria related to use of an intrauterine contraceptive device with progesterone local therapy also includes women who were using this at the time of menopause.<br>Inclusion criteria updated to include at least 2, painful hand joints of any type (interphalangeal or base of thumbs). Patients with base of thumb OA only do not need to meet the American College of Rheumatology criteria.<br>Broadening of inclusion criteria to consider 'typical' average hand pain as well as average hand pain reported in the last 7 days at the screening visit.<br>Changes to recruitment strategies (8.1.1.):<br>• Broadening the method by which the GP database search will be carried out to include SMS messaging as well as posting study details to those identified by a database search.<br>• Addition of Fortius Clinic as a secondary care Participant Identification Centre.<br>• The addition of community advertising to the recruitment strategy (posters, flyers, social media).<br>A further stratification group (base of thumb OA only) has been added to Section 8.5 to align with the change to inclusion criteria.<br>Statistics section (11.1) updated to reflect the three stratification groups.
2	2·0	20Jun 2019	The Inclusion criteria have been updated to include women who are using an intrauterine contraceptive device with progesterone local therapy (such as Mirena). These women, will need to meet the same inclusion criteria as those women who have undergone a hysterectomy because this group of women do not have regular periods and therefore 12 months of spontaneous amenorrhea is not a reliable method for determining menopause.<br>Blood volume at the Screening Visit increased from 20 ml to 25 ml. The hormone testing needs to be in a separate 5 ml SST II Gold top tube to the Autoantibody testing and 20 ml is not sufficient for this.<br>The optional study (to provide an additional sample of blood and urine) has been removed from the protocol because it comes under a separate Research Ethics Committee approval.<br>Section 10.2.1 has been updated detailing the process that should be followed if a participant experiences vaginal bleeding. This change is necessary because those participants who are on placebo and experience breakthrough bleeding should be referred for urgent investigation of post-menopausal bleeding as per NICE guidelines.<br>Section 8.5 (code breaking) has been modified to limit unnecessary unblinding of the Investigators. Also, in circumstances where unblinding occurs via local Trials Pharmacy, only an unblinding request form will be required to be sent to the pharmacist and not a log. The unblinding log will instead be updated by the central study team once the record of unblinding form has been received (this form does not reveal the treatment allocation unlike the unblinding request form).<br>Frimley Health NHS Foundation Trust has been added as a participant identification centre.<br>The list of centres where a participant at Faringdon may have their X-ray has been expanded to mention that the X-ray can be performed at a choice of several nearby centres.<br>Oxford Brookes hand clinic as a method of recruitment has been removed from because this clinic is no longer running.<br>References to Arthritis Research UK Centre for OA Pathogenesis have been changed to Centre for OA Pathogenesis Versus Arthritis in line with Arthritis Research UK's new name 'Versus Arthritis'.<br>Physical Examination has been added Visit 3 in the table in Appendix B of the protocol to be consistent with Section 8.7 of the protocol.<br>The study medication, CE-Bazedoxifene, has not been ordered via the NHS Supply chain, Section 9.3 of the protocol has been updated to reflect this.

Supplementary Table 1. Protocol amendment history for the study
Amendment 1 was a minor amendment which did not include amendment to protocol, therefore is not listed. The protocol was published following amendment 4 and therefore does not include amendment 5-8 (Marian et al., 2021).

**Inclusion Criteria**	• Able to give informed written consent<br>• Female, aged 40-65 years old<br>• In those with an intact uterus: At least 12 months of spontaneous amenorrhea (without any menstrual bleeding in last 12 months) and last menstrual period not more than 10 years ago<br>• In those who have undergone hysterectomy or are/were using an intrauterine contraceptive device with progesterone local therapy (such as Mirena): Follicle stimulating hormone (FSH) ≥30 milli-International Units per millilitre (mIU/ml) on screening blood test AND a history of menopausal symptoms in the last 1 to 10 years, in keeping with appropriate timing of menopausal status<br>• Hand pain, aching or stiffness on most days in the last 3 months<br>• At least 2, painful hand joints of any type (interphalangeal joints (IPJ) or base of thumbs)<br>• Fulfils American College of Rheumatology clinical diagnostic criteria for hand OA (Altman et al., 1990) (3 or more of following):<br><br>o Hard tissue enlargement of 2 or more of the following joints: 2nd or 3rd distal interphalangeal joints (DIPJ), 2nd or 3rd proximal interphalangeal joints (PIPJ), first carpometacarpal joints (CMCJ)<br>o Hard tissue enlargement of 2 or more of the DIPJs<br>o Less than 3 swollen metacarpophalangeal joints (MCPJ)<br>o Deformity of at least one of the joints listed in first point<br>o OR, for those with base of thumb osteoarthritis only not fulfilling these criteria, has clinical symptoms and examination findings consistent with base of thumb osteoarthritis.<br><br>• Hand pain has not responded adequately to NICE core guidance for management of OA, including the use of paracetamol or non-steroidal anti-inflammatory drug gel, except where there is contraindication or intolerance<br>• Average hand pain is reported as typically more than 4 out of 10 in severity, OR average hand pain in the last 7 days of 4/10 or more on a visual analogue scale<br>• In the Investigator's opinion, is able and willing to comply with all study requirements
**Exclusion Criteria**	The participant may not enter the study if ANY of the following apply:<br>• Other cause of hand pain, including inflammatory arthritis, connective tissue disorder, chronic pain or alternative clinical diagnosis such as tenosynovitis or carpal tunnel syndrome<br>• Pregnancy or breast feeding, or risk of this during study<br>• Use of one or more prohibited treatments within specified timeframe, or not willing to avoid treatment for the duration of the study:<br><br>o Oral contraceptive pill, or systemic Hormone Replacement Therapy (HRT) within the last 6 months[ǂ]<br>o Anti-estrogen medication within the last 6 months<br>o Oral, intramuscular or intraarticular steroid within the last 3 months<br>o Intraarticular hyaluronan to a hand joint within the last 6 months<br>o Initiation of new oral analgesia within the last 4 weeks<br>o Initiation of glucosamine, chondroitin, hand exercises or other relevant non-pharmacological therapy within the last 6 weeks<br>o Hand surgery within the last 6 months, or planned within the next 6 months<br>o Medications likely to increase hepatic metabolism of study medication, including:<br><br>▪ St. John's Wort<br>▪ Anti-convulsants (phenobarbital, phenytoin, carbamazepine, lamotrigine)<br>▪ Some anti-infectives (rifampicin, rifabutin, nevirapine, efavirenz, ritonavir and nelfinavir)<br><br>• Presence of one or more medical contraindications to the use of systemic HRT:<br><br>o In those aged 40-45 years, FSH <30 mIU/ml on screening blood test, i.e. non- confirmatory of menopausal status<br>o Any history of breast, endometrial, ovarian or skin cancer<br>o Any other history of other cancer within 5 years (except treated Basal Cell Carcinoma [BCC])<br>o Relevant breast issue on routine national breast screening in prior 3 years<br>o Undiagnosed genital bleeding, or untreated endometrial hyperplasia, active uterine fibroids or endometriosis<br>o Active or past history of venous thromboembolism (VTE) (including deep venous thrombosis, pulmonary embolism and retinal vein thrombosis), or at high risk of VTE (such as known thrombophilic disorders (such as Protein C, S or anti-thrombin deficiency) or presence of a strong family history of VTE[¥])<br>o Active or past history of arterial thrombo-embolic disease (such as myocardial infarction, angina or stroke) or strong family history of stroke[¥])<br>o Clinically significant immobility<br>o Migraine or active epilepsy<br>o Uncontrolled hypertension (or diastolic pressure greater than 90 mmHg or systolic pressure greater than 145 mmHg at Screening Visit)<br>o Uncontrolled diabetes mellitus or uncontrolled hypertriglyceridaemia<br>o Body Mass Index greater than 30<br>o Active malabsorption syndrome or clinically significant small bowel disease<br>o Acute liver disease, clinically significant abnormal liver function, active gallbladder disease or porphyria<br>o Clinically significant renal impairment<br>o Intolerance to lactose, fructose or glucose (including galactose intolerance, lactase deficiency, fructose intolerance, glucose-galactose malabsorption or sucrase-isomaltase insufficiency)<br>o Known sensitivity to either conjugated equine estrogens, bazedoxifene or the combination

	• Any other significant or uncontrolled disease or disorder which, in the opinion of the Investigator, may either put the participants at risk because of participation in the study, or may influence the result of the study, or the participant's ability to participate in the study<br>• Participants who have participated in another research trial involving an investigational product in the past 8 weeks

Supplementary Table 2. Full eligibility criteria for the study

[ǂ]Use of an intrauterine contraceptive device with progesterone local therapy (Mirena) or vaginal topical estrogen use (known low systemic absorption) are not exclusions to participation.
[¥]women with a first degree relative with a history of venous thromboembolism (VTE), or other strong family history of VTE at the Investigators' discretion.

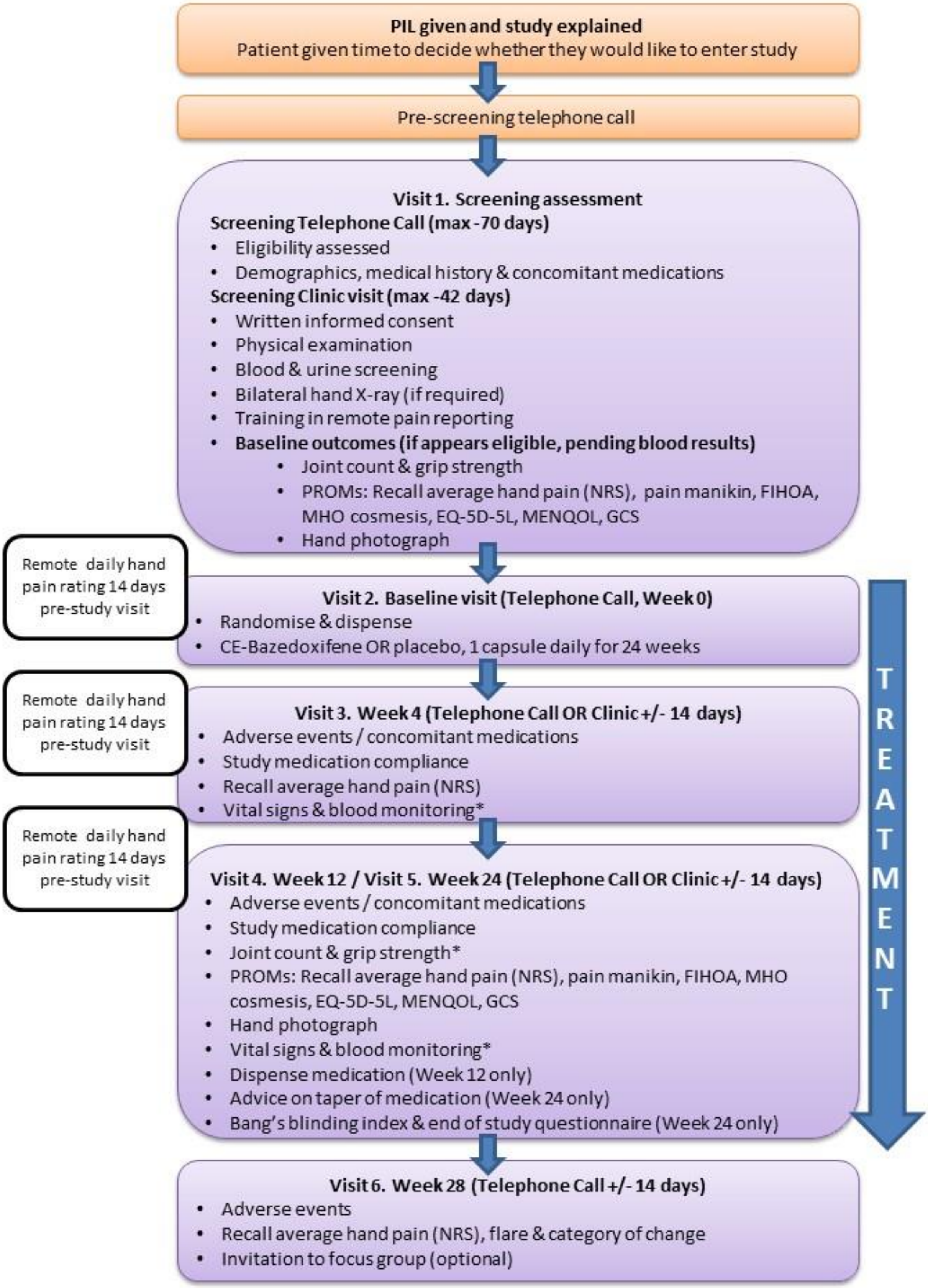

Supplementary Figure 1. Study visit schedule flow chart

The study visit schedule was changed post-pandemic following an amendment to reduce face-to-face contact at study visits (see Table S1). This revised study schedule was implemented from August 2020 to August 2021. The pre-

pandemic study visit schedule was followed from May 2019 to July 2020 and can be found in the published protocol (Marian et al., 2021).

* Procedures not performed when the study visit was remote. If medically required, blood pressure readings were taken by the participant at home or at their General Practice.

PIL, Patient Information Leaflet; PROMs, Patient Reported Outcome Measures; NRS, Numerical Rating Scale; FIHOA, Functional Index for Hand Osteoarthritis; MHO, Michigan Hand Outcomes; MENQOL, Menopause specific Quality of Life questionnaire; GCS, Greene Climacteric Scale; CE-Bazedoxifene, Conjugated Estrogens-Bazedoxifene

Source	Number of enquiries/ clinician referrals	Number of randomisations	% Randomised from total
Online local newspaper	11	2	18
Word of mouth	25	4	16
Website	62	7	11
Poster/flyer	50	4	8
SMS	58	5	9
Clinician	59	4	7
GP surgery/poster	39	2	5
Newsletter	16	0	0
Social Media	12	0	0
Magazine/Radio	9	0	0
Invitation letter	7	0	0
Staff bulletin	4	0	0
Not known	82	0	0
**Total**	**434**	**28**	**7**

Supplementary Table 3. Rates of recruitment from different sources

GP, General Practice; SMS, Short Message Service

	NOC	CXH	WHMP	Overall
Total randomisations	14	5	9	**28**
Total months site open	15	12	13	**15**
Average randomisations, per month open	1	0·4	0·7	**2**

Supplementary Table 4. Randomisations per month, by site
All sites and recruitment were in England, UK.

NOC, Nuffield Orthopaedic Centre, Oxford University Hospitals NHS Foundation Trust, Oxford, UK; CXH, Charing Cross Hospital, Imperial College Healthcare NHS Trust, London, UK; WHMP, White Horse Medical Practice, Oxfordshire, UK.

Recruitment Rate		Screened<br>N	Randomised<br>N	Recruitment Rate<br>(95% CI)
**Overall**		35	28	80 % (63%, 92%)
	**Telephone Screening**	**Face-to-Face Screening**	**Randomised**	**Recruitment Rate<br>(95% CI)**
Pre-amendments	-	8	5	63% (24%, 91%)
Post-amendment	27	25	23	85% (66%, 96%)

Supplementary Table 5. Recruitment rates pre/post amendments 4 and 5
Details of amendments 4 and 5 due to low recruitment and to the pandemic respectively can be found in Table S1.

CI, confidence interval

Hand pain method	Baseline	Week 24	Retention Rate (95% CI)[1]
Recall mean hand pain	28	28	100% (100%, 100%)
Daily mean hand pain[2]	28	28	100% (100%, 100%)
Number of daily hand pain scores recorded[#]			
Median (IQR)	*14 (13 - 14)*	*14 (13 - 14)*	
Min-Max	*12-14*	*12-14*	

Supplementary Table 6. Retention rates, based on collection of mean hand pain

[1] Number who provided mean hand pain that were included in the analysis
[2] One participant completed remote hand pain via paper diaries and 27 participants completed hand pain electronically
[#]Maximum possible number of daily hand pain scores is 14.

IQR, interquartile range; CI, confidence interval

A

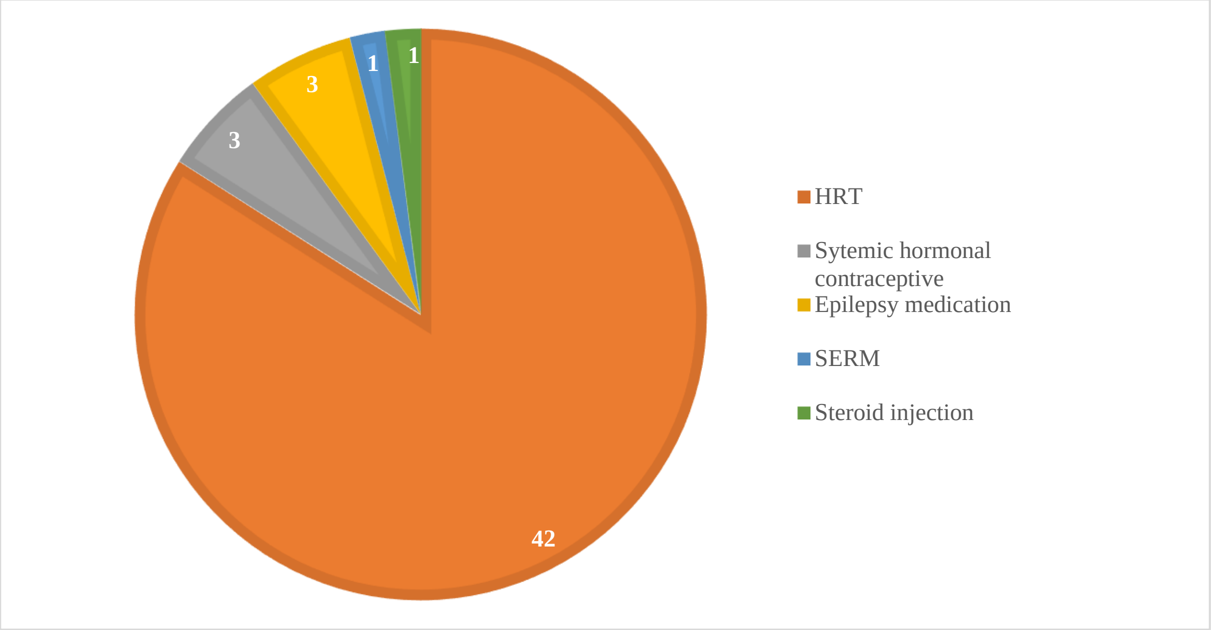

B

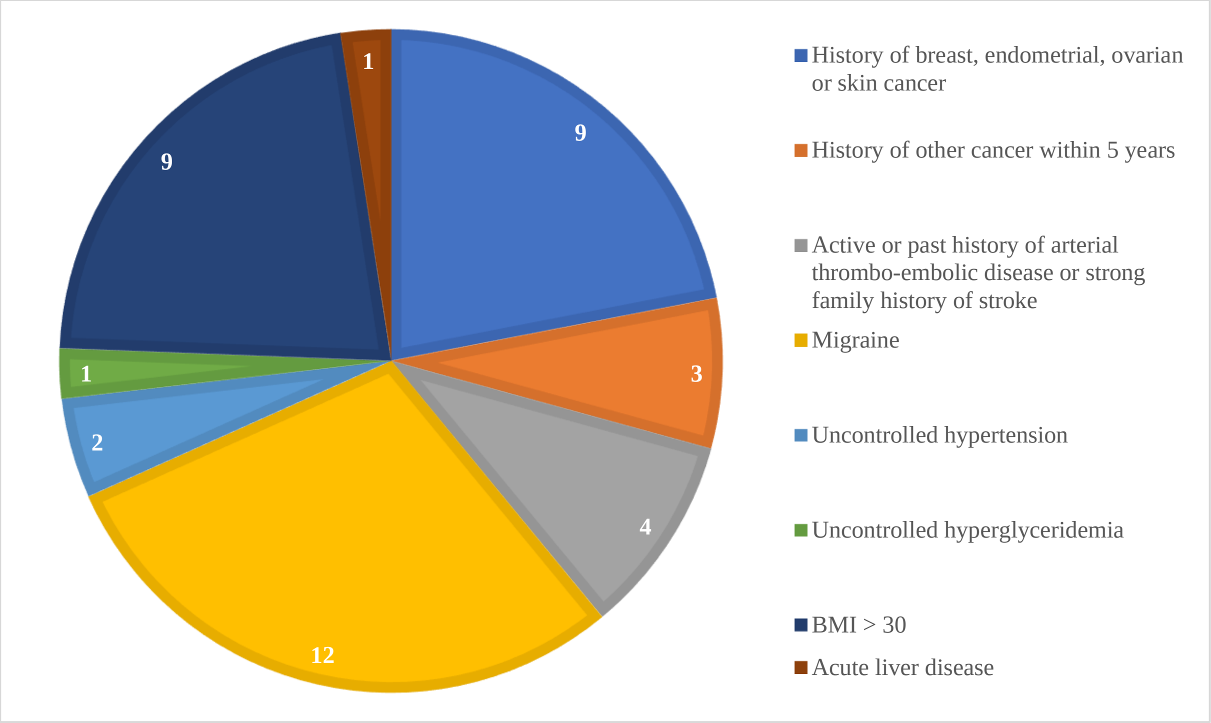

Supplementary Figure 2. Reasons for ineligibility (medication and medical contraindications)
(A) Reasons for ineligibility due to contraindicated medications are shown, by contraindicated drug class. Numbers of individuals in each category are given, n=50. (B) Reasons for ineligibility due to medical contraindications are shown. Number of individuals in each category are given, n=41. Where more than one contraindication was identified, the first reached on the exclusion criteria is shown.

SERM, Selective Estrogen Receptor Modulator; BMI, Body Mass Index

Participant	Treatment	Days receiving study medication	Reason
A*	Placebo	42	Participant decision - Adverse Event
B Ɫ	Placebo	174	Investigator decision - New ineligibility and new significant medical condition
C Ɫ	Placebo	180	Investigator decision - Unblinding for safety reason

Supplementary Table 7. Withdrawals from treatment during the study
*This participant was withdrawn from study medication before week 12 but continued follow-up
Ɫ Participants withdrew at the point of weaning, week 24

	CE-Bazedoxifene<br>n=14	Placebo<br>n=14	Total<br>n=28
**Participants with protocol deviations (n,%)**	4, (29%)	4, (29%)	8 (29%)
**Protocol deviations (n)**	4	5	9
**Deviation Impact**			
Accuracy of study results[1]	0 (%)	1 (20%)	1 (11%)
No impact on study results or participant safety	4 (100%)	4 (80%)	8 (89%)

Supplementary Table 8. Protocol deviations during the study

[1]One protocol deviation was considered important (that is, that it could have led to an impact on study results or participant safety): between screening and re-screening the stratification factor of Interphalangeal Joint (IPJ) group for one individual changed from base of thumb OA to IPJ plus base of thumb OA. This could not be changed in the randomisation programme, and the participant was randomised with the incorrect stratification factor.

CE-Bazedoxifene, Conjugated Estrogens-Bazedoxifene

	CE-Bazedoxifene n=14	Placebo n=14
**Participant Response**		
Strongly believe I received the active treatment	1 (7%)	0 (%)
Somewhat believe I received the active treatment	5 (36%)	2 (14%)
Somewhat believe I received placebo	4 (29%)	3 (21%)
Strongly believe I received placebo	3 (21%)	6 (43%)
Don't Know	1 (7%)	2 (14%)
Unblinded to their treatment allocation [1]	*0 (%)*	*1 (7%)*
**Participant BBI**[1]	**-0·11 (-0·40, 0·18)**	**0·50 (0·25, 0·75)**
**Investigator Response**		
Strongly believe participant had the active treatment	0 (%)	0 (%)
Somewhat believe the participant received the active treatment	0 (%)	2 (14%)
Somewhat believe the participant received placebo	0 (%)	1 (7%)
Strongly believe the participant received placebo	0 (%)	0 (%)
Don't Know	14 (100%)	9 (64%)
Unblinded to the participant's treatment allocation[2]	*0 (%)*	*2 (14%)*
**Investigator BBI**	**0 (0,0)**[3]	**-0·04 (-0·16, 0·08)**

Supplementary Table 9. Participant and Investigator response to blinding questions at week 24 and corresponding Bang's Blinding Index

For those who remained blinded at the 24 week visit, the time of being asked what treatment allocation they thought they were on, the Bangs Blinding Index (BBI) seeks to quantify what proportion of participants correctly guess their allocation beyond chance. BBI scores range from -1 (all participants guess their allocation incorrectly indicating opposite guessing which could be related to blinding), 0 (perfect blinding) to 1 (all participants guess their allocation correctly indicating complete lack of blinding) (Chen Jiefeng, 2008, Bang, Ni & Davis, 2004).

[1] One participant was unblinded before answering this question and is excluded from the index calculation

[2] The investigator was unblinded twice in the placebo arm and these proportions were excluded from the index calculation

[3] The investigator did not know the allocation for all 14 of the participants on active treatment

CE-Bazedoxifene, Conjugated Estrogens-Bazedoxifene

	CE-Bazedoxifene (n=14)	Placebo (n=14)	Total (n=28)
**Blinded throughout study**	**13 (93%)**	**10 (71%)**	**23 (82%)**
**Unblinded**	**1 (7%)**	**4 (29%)**	**5 (18%)**
Intentional - Participant, Site staff and Central Study Team pre-week 24	0 (%)	1 (7%)	1 (4%)
Intentional - Participant, Site staff and Central Study Team post-week 24	0 (%)	2 (14%)	2 (7%)
Accidental - Member of Central Study Team only pre-week 24	1 (7%)	0 (%)	1 (4%)
Accidental – Site-staff only, post-week 24	0 (%)	1 (7%)	1 (4%)

Supplementary Table 10. Accidental and intentional unblinding

There were five participants' allocations that became unblinded to members of the study team; three of these allocations were unblinded to the participant also. Of these, three were incidents of intentional unblinding for safety, all in the placebo arm. There were two accidental unblinding incidents, only one of which affected site staff, and occurred following the collection of week 24 outcomes.

	Baseline n (%)		Week 24 n (%)	
	CE-Bazedoxifene	Placebo	CE-Bazedoxifene	Placebo
Recall Mean Hand Pain	14 (100)	14 (100)	14 (100)	14 (100)
Daily Mean Hand Pain	14 (100)	14 (100)	14 (100)	14 (100)
EQ-5D-5L Utilities	14 (100)	14 (100)	14 (100)	14 (100)
EQ-5D-5L VAS	14 (100)	14 (100)	14 (100)	14 (100)
Michigan Hand Outcome (Cosmesis)	14 (100)	14 (100)	14 (100)	14 (100)
FIHOA	13 (93)[1]	14 (100)	14 (100)	14 (100)
Greene Climacteric Scale	13 (93)[1]	13 (93)[1]	14 (100)	13 (93)[1]
MENQOL	11 (79)[1]	12 (86)[1]	14 (100)	13 (93)[1]

Supplementary Table 11. Completeness of patient reported outcome measures at Baseline and Week 24
The number (n) and percentage (%) of completeness of items from outcome measures are shown.
[1]Missing items were subsequently imputed and included in analysis.

CE-Bazedoxifene, Conjugated Estrogens-Bazedoxifene; FIHOA, Functional Index for Hand Osteoarthritis; MENQOL, Menopause specific Quality of Life questionnaire

A

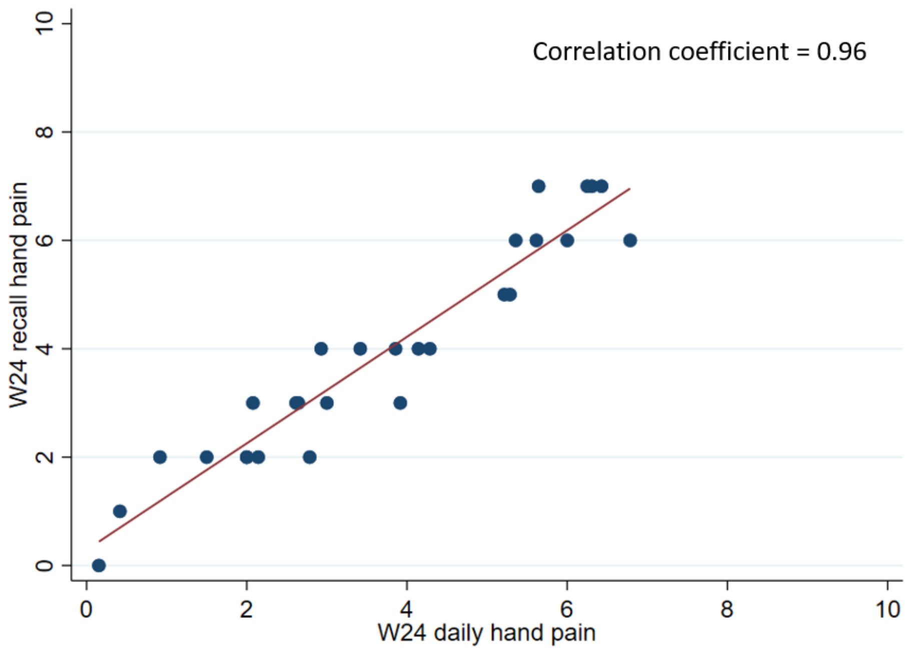

B

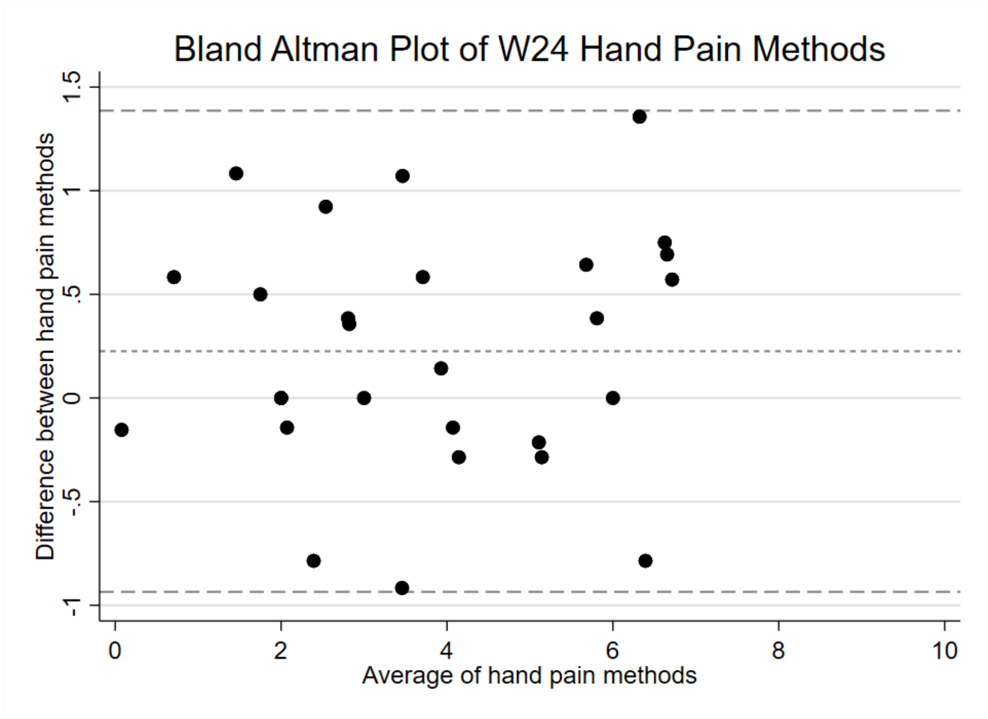

Supplementary Figure 3. Comparison of two methods of rating mean hand pain during the study
Comparison of participant data for two methods of rating mean hand pain over preceding 14 days on numerical rating scale, by recall and by daily rating is compared at Week 24 visit (W24).

(A) Week 24 hand pain measurements: scatter plot of individuals' ratings by the two methods. Pearson's r correlation coefficient is shown. (B) Bland Altman Plot of week 24 hand pain measurements by the two methods.

	CE-Bazedoxifene (n=14) N, Mean (SD)	Placebo (n=14) N, Mean (SD)	Treatment effect (95% Confidence Interval)[1]
**EQ-5D utility**[2]			0·0629 (0·0334, 0·0924)
Baseline	14, 0·707 (0·138)	14, 0·747 (0·175)	
Week 12	14, 0·755 (0·059)	14, 0·705 (0·141)	
Week 24	14, 0·683 (0·141)	14, 0·750 (0·086)	
**EQ-5D VAS**[3]			11·5 (8·3, 14·7)
Baseline	14, 77·0 (13·7)	14, 80·1 (9·4)	
Week 12	14, 78·8 (12·8)	14, 69·3 (18·3)	
Week 24	14, 78·3 (11·9)	14, 76·1 (15·7)	
**FIHOA**[4]			-1·0 (-4·5, 2·5)
Baseline[5]	14, 7·1 (4·9)	14, 6·4 (5·5)	
Week 12[6]	14, 6·7 (4·6)	14, 7·0 (5·6)	
Week 24	14, 6·9 (5·5)	14, 5·7 (5·7)	

Supplementary Table 12. Health quality of life and hand function in participants over time
This analysis was performed for the Intention-to-treat population using available cases.

[1] Modelled with a mixed effect model with time treatment interaction and time as a categorical variable, adjusted for baseline values, minimisation factors (type of painful hand joints) with cluster robust standard errors for sites.
[2] EQ-5D utility scores range from -0·594 to 1 with 1 representing perfect health and 0 equivalent to death.[3] EQ-5D VAS scores range scores from 0 (worst possible health) to 100 (best possible health). (van Hout et al., 2012, Janssen et al., 2013)
[4] FIHOA, functional Index for Hand OA, lower scores indicate better hand function (Dreiser et al., 1995)
[5] One participant missed the item for key lock which was imputed as the mean of available answers
[6] One participant missed the item for writing and one participant missed the item clench fist. Both missing items were imputed as the mean of available answers

CE-Bazedoxifene, Conjugated Estrogens-Bazedoxifene; VAS, Visual Analogue Scale

	CE-Bazedoxifene (n=14) N, Mean (SD) *Variance of three measures, Median (IQR)*	Placebo (n=14) N, Mean (SD) *Variance of three measures, Median (IQR)*	Treatment Effect (95% Confidence Interval)[1]
**Grip strength (right), kg**			0·57 (-2·22, 3·37)
**Baseline**	14, 20·4 (6·8) *2·9 (2·0, 4·7)*	14, 19·2 (8·0) *1·9 (0·36, 6·4)*	
**Week 24**	13, 21·6 (4·5) *1·6 (0·66, 1·96)*	12, 21·6 (7·3) *1·37 (0·51, 4·93)*	
**Grip strength (left), kg**			-0·92 (-4·43, 2·59)
**Baseline**	14, 19·6 (6·3) *5·0 (3, 8·7)*	14, 17·7 (6·6) *2·9 (0·86, 6·54)*	
**Week 24**	13, 19·2 (5·0) *2·36 (1·00, 4·63)*	12, 20·3 (6·0) *2·07 (1·57, 4·54)*	

Supplementary Table 13. Grip strength in participants over time
A mean of three attempts for grip strength in each hand was measured by dynamometer in kilograms (kg). (Mathiowetz et al., 1984)
[1] ANOVA treatment effect at week 24 adjusted for baseline values and minimisation factors (type of painful hand joints and site).

CE-Bazedoxifene, Conjugated Estrogens-Bazedoxifene; IQR, interquartile range

	Baseline		Week 24		Incident Rate Ratio
	CE-Bazedoxifene (n=14)	Placebo (n=14)	CE-Bazedoxifene (n=14)	Placebo (n=14)	(95% CI)[1]
	N, Median (IQR)	N, Median (IQR)	N, Median (IQR)	N, Median (IQR)	
**Painful Joints**					
DIPJ[2]	11, 2 (1,4)	11, 4 (1,6)	8, 1 (0,3)	8, 2 (0,6)	0.44 (0.19, 1.04)
PIPJ[3]	13, 3 (2,6)	10, 3 (0,6)	9, 2 (0,4)	8, 5 (0,9)	0.49 (0.24, 1.02)
Finger DIPJ/PIPJ[4]	14, 5 (1,9)	12, 7 (2,8)	8, 3 (0,7)	10, 7 (2,11)	0.40 (0.18, 0.86)
Thumb IPJ/CMCJ/STT[5]	12, 5 (3,6)	12, 4 (3,6)	11, 4 (2,4)	10, 3 (1,5)	0.83 (0.51, 1.35)
All joints[6]	14, 10 (8,15)	14, 12 (6,15)	13, 6 (4,7)	12, 9 (4,15)	0.63 (0.38, 1.02)
**Swollen Joints**					
DIPJ[2]	5, 0 (0,1)	6, 0 (0,1)	5, 0 (0,1)	6, 1 (0,2)	0.49 (0.19, 1.26)
PIPJ[3]	11, 2 (1,3)	7, 1 (0,2)	7, 1 (0,2)	7, 1 (0,2)	0.62 (0.25, 1.52)
Finger DIPJ/PIPJ[4]	9, 2 (0,3)	8, 1 (0,2)	7, 1 (0,2)	9, 2 (1,3)	0.50 (0.21, 1.15)
Thumb IPJ/CMCJ/STT[5]	8, 1 (0,1)	5, 0 (0,2)	7, 1 (0,1)	6, 1 (0,1)	1.29 (0.51, 3.25)
All joints[6]	13, 2 (1,5)	10, 2 (0,3)	11, 1 (1,4)	10, 3 (1,4)	0.65 (0.34, 1.23)
**Tender Joints**					
DIPJ[2]	9, 2 (0,6)	10, 3 (0,3)	9, 1 (0,4)	6, 1 (0,2)	1.00 (0.47, 2.13)
PIPJ[3]	11, 4 (1,6)	12, 2 (1,4)	9, 2 (0,2)	7, 1 (0,4)	0.51 (0.24, 1.08)
Finger DIPJ/PIPJ[4]	12, 4 (2,10)	11, 4 (2,7)	10, 2 (1,6)	8, 3 (0,4)	0.68 (0.34, 1.34)
Thumb IPJ/CMCJ/STT[5]	10, 3 (0,4)	12, 3 (1,6)	8, 3 (0,4)	8, 2 (0,4)	1.35 (0.75, 2.43)
All joints[6]	13, 7 (5,12)	14, 8 (4,13)	13, 5 (3,11)	11, 4 (3,7)	0.84 (0.53, 1.33)

Supplementary Table 14. Tender, painful and swollen joints over time

Joint score categories listed were calculated as the sum of scores from individual joints within these groups. Higher scores indicate higher counts of painful, swollen or tender joints in these groups.

[1] Modelled with negative binomial adjusting for baseline values and stratification factors. Incident rate ratio (IRR) <1 indicates number of painful, swollen or tender joints is lower in the CE-Bazedoxifene group. Incident rate ratio (IRR) >1 indicates number of painful, swollen or tender joints is greater in the CE-Bazedoxifene group. IRR = 1 or 95% CI contains 1 indicates no treatment difference.

[2] DIPJ, Distal interphalangeal joints, score ranges from 0 to 8

[3] PIPJ, Proximal interphalangeal joints, score ranges from 0 to 10

[4] Finger DIPJ/PIPJ, score ranges from 0 to 16

[5] Thumb IPJ (Interphalangeal joints)/CMCJ, Carpometacarpal joint) /STT, Scaphotrapeziotrapezoid joint, score ranges from 0 to 8

[6] All joints, score ranges from 0 to 32

CE-Bazedoxifene, Conjugated Estrogens-Bazedoxifene; IQR, interquartile range; CI, confidence interval

	CE-Bazedoxifene (n=14) *N (%)*	Placebo (n=14) *N (%)*	Odds Ratio (95% CI)[1]
**Pain in both hands**[2]			0·78 (0·11, 5·61)
Baseline	13 (93)	12 (86)	
Week 24	11 (79)	11 (79)	
**Pain at any site other than hand**[3]			0·80 (0·10, 6·67)
Baseline	13 (93)	11 (79)	
Week 24	11 (79)	10 (71)	
**Pain at 4 or more sites**[4]			0·74 (0·16, 3·39)
Baseline	7 (50)	8 (57)	
Week 24	5 (36)	6 (43)	
	Median (IQR)	*Median (IQR)*	*Incident Rate Ratio[6] (95% CI)*
**Total sites,**[5]			*0.89 (0.53, 1.48)*
Baseline	*4 (3,7)*	*4 (2,6)*	
Week 24	*3 (2,4)*	*3 (1,5)*	

Supplementary Table 15. Pain and function outcomes in participants over time

[1] Odds ratio of 1 and confidence interval that contains 1 indicates no treatment difference. Odds ratio <1 indicates less pain in CE-Bazedoxifene compared to placebo
[2] Participants reporting pain in both hands. Odds ratio from logistic regression adjusting for minimisation factors only, model could not adjust for baseline scores because of collinearity
[3] Participants reporting pain at any other non-hand sites: neck, shoulders, elbows, back, hips, knees or feet. Odds ratio from logistic regression adjusting for baseline and minimisation factors
[4] Participants reporting pain at 4 or more sites. Odds ratio from logistic regression adjusting for treatment only. Model could not adjust for baseline scores or minimisation factors because of collinearity
[5] Count of the total number of sites with pain
[6] Treatment effect modelled as negative binomial adjusting for baseline and minimisation factors. A coefficient <1 indicates the number of painful sites is lower for those receiving CE-Bazedoxifene compared to placebo. A coefficient of 1 and confidence interval that contains 1 indicates no treatment difference.

CE-Bazedoxifene, Conjugated Estrogens-Bazedoxifene; IQR, interquartile range; CI, confidence interval

Michigan Hand Outcome: Aesthetics Questions[1]	CE-Bazedoxifene (n=14) N, Mean (SD)	Placebo (n=14) N, Mean (SD)	Treatment Effect (95% Confidence Interval)[2]
**Aesthetics Right**			
Baseline	14, 65·2 (23·2)	14, 63·8 (28·1)	3·7 (-1·9, 9·3)
Week 12	14, 65·6 (27·4)	14, 60·7 (24·7)	
Week 24	14, 62·9 (26·2)	14, 63·4 (26·7)	
**Aesthetics Left**			
Baseline	14, 64·3 (25·8)	14, 65·2 (24·1)	5·0 (1·8, 8·2)
Week 12	14, 69·6 (25·1)	14, 65·6 (23·5)	
Week 24	14, 69·6 (24·7)	14, 66·1 (27·6)	

Supplementary Table 16. Joint appearance measured by Michigan Hand Outcome questionnaire (aesthetic/cosmesis questions only) in participants over time

[1] Range from 0 to 100 where higher scores indicate better hand appearance (Scoring the MHQ, Chung et al., 1998)
[2] Mixed effect model with time treatment interaction and time as a categorical variable, adjusted for baseline values, minimisation factors (type of painful hand joints) with cluster robust standard errors for sites

CE-Bazedoxifene, Conjugated Estrogens-Bazedoxifene

	CE-Bazedoxifene (n=14) N, Mean (SD)	Placebo (n=14) N, Mean (SD)	Treatment Difference (95% Confidence Interval)[1]
**MENQOL[2]**			
**Vasomotor Domain**			-0·124 (-0·606, 0·358)
Baseline	14, 2·45 (1·80)	14, 3·26 (1·63)	
Week 12	14, 2·17 (1·38)	14, 2·88 (1·62)	
Week 24	14, 2·29 (1·96)	14, 3·12 (1·44)	
**Psychosocial Domain**			-0·199 (-0·757, 0·359)
Baseline	14, 2·83 (1·43)	14, 2·73 (1·16)	
Week 12	14, 2·68 (1·48)	14, 2·81 (1·36)	
Week 24	14, 2·99 (1·33)	14, 2·55 (1·14)	
**Physical Domain**			0·059 (0·014, 0·104)
Baseline	14, 2·87 (0·75)	14, 3·23 (1·04)	
Week 12	14, 2·69 (1·13)	14, 2·86 (0·93)	
Week 24	14, 3·13 (0·93)	14, 2·97 (1·18)	
**Sexual Domain**			-0·105 (-1·073, 0·863)
Baseline	14, 2·81 (2·00)	14, 4·24 (2·48)	
Week 12	14, 2·50 (2·05)	14, 3·81 (2·49)	
Week 24	14, 2·67 (1·98)	14, 3·98 (2·43)	
**Overall MENQOL Score**			-0·042 (-0·164, 0·079)
Baseline	14, 2·74 (0·90)	14, 3·37 (0·95)	
Week 12	14, 2·51 (1·01)	14, 3·09 (1·05)	
Week 24	14, 2·77 (1·06)	14, 3·15 (1·11)	
**GCS[3]**			
**Anxiety Domain**			-1·027 (-2·589, 0·534)
Baseline	14, 3·86 (2·11)	14, 3·64 (1·22)	
Week 12	14, 3·29 (3·29)	14, 4·07 (2·59)	
Week 24	14, 3·57 (2·59)	14, 3·43 (1·79)	
**Depression Domain**			-0·863 (-1·696, -0·031)
Baseline	14, 3·14 (2·21)	14, 2·50 (1·91)	
Week 12	14, 2·29 (1·77)	14, 2·71 (2·13)	
Week 24	14, 3·29 (2·05)	14, 2·50 (2·47)	
**Somatic Domain**			-1·049 (-2·461, 0·363)
Baseline	14, 2·93 (1·33)	14, 2·71 (1·38)	
Week 12	14, 2·57 (1·60)	14, 3·43 (2·59)	
Week 24	14, 3·50 (1·79)	14, 3·14 (3·16)	
**Vasomotor Domain**			0·158 (-0·250, 0·565)
Baseline	14, 1·36 (1·60)	14, 2·14 (1·88)	
Week 12	14, 1·36 (1·55)	14, 1·71 (1·44)	
Week 24	14, 1·07 (1·64)	14, 1·86 (1·29)	
**Sexual Domain**			-0·331 (-0·487, -0·174)
Baseline	14, 0·93 (1·14)	14, 1·50 (1·16)	
Week 12	14, 0·86 (1·17)	14, 1·64 (1·22)	
Week 24	14, 0·71 (0·83)	14, 1·64 (1·22)	
**Overall GCS Score**			-3·066 (-5·720, -0·412)
Baseline	14, 12·21 (5·10)	14, 12·50 (5·06)	
Week 12	14, 10·36 (6·80)	14, 13·57 (6·12)	
Week 24	14, 12·14 (6·30)	14, 12·57 (7·47)	

Supplementary Table 17. Menopause symptoms measured by MENQOL and Greene Climacteric Scale in participants over time

Missing items for either score were imputed as the mean of the responses from that participant to items available for that domain if 50% of items were available.

[1] Global treatment difference was calculated with a mixed effect model with time treatment interaction and time as a categorical variable, adjusted for baseline values, minimisation factors (type of painful hand joints) and cluster robust standard errors for sites.

[2] MENQOL modified questionnaire consists of 32 items divided into four domains. For each item, women are asked whether she experienced the item in the previous month. If no, she receives a score of 1. If yes, she indicates how bothered she is and is given a score ranging from 2 'yes - not at all bothered' to 8 'yes - extremely bothered'. Each of the domain score is the mean of the item scores forming that domain. Higher MENQOL scores indicate worse symptoms.

[3] GCS is a 21-item validated questionnaire that measures menopausal symptoms on a 4-point Likert scale (0 = “not at all” to 3 = “extremely”) and one sexual function probe. The total score is the sum of the scores obtained for each domain and ranges from 0 to 63 points. Higher scores indicate more bothersome symptoms.

(Radtke, Terhorst & Cohen, 2011, Lewis, Hilditch & Wong, 2005, Burbos, Morris, 2010, Greene, 1998)

CE-Bazedoxifene, Conjugated Estrogens-Bazedoxifene; MENQOL, Menopause specific Quality of Life questionnaire; GCS, Greene Climacteric Scale

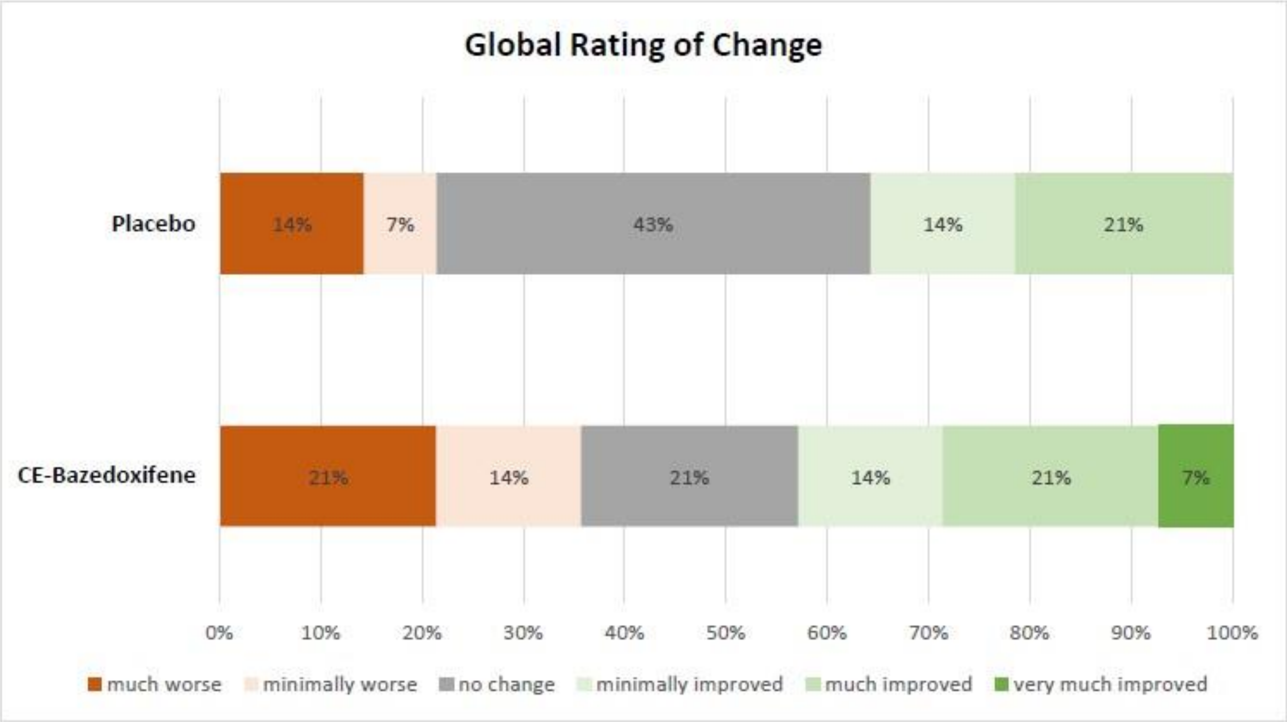

Supplementary Figure 4. Patient reported Global impression of change at week 24 visit, by treatment arm
Participants were asked, "Since the start of the study, my overall status is: Very much improved, much improved, minimally improved, no change, minimally worse, much worse or very much worse". No-one chose "very much improved" on placebo. One participant is 7%.

CE-Bazedoxifene, Conjugated Estrogens-Bazedoxifene

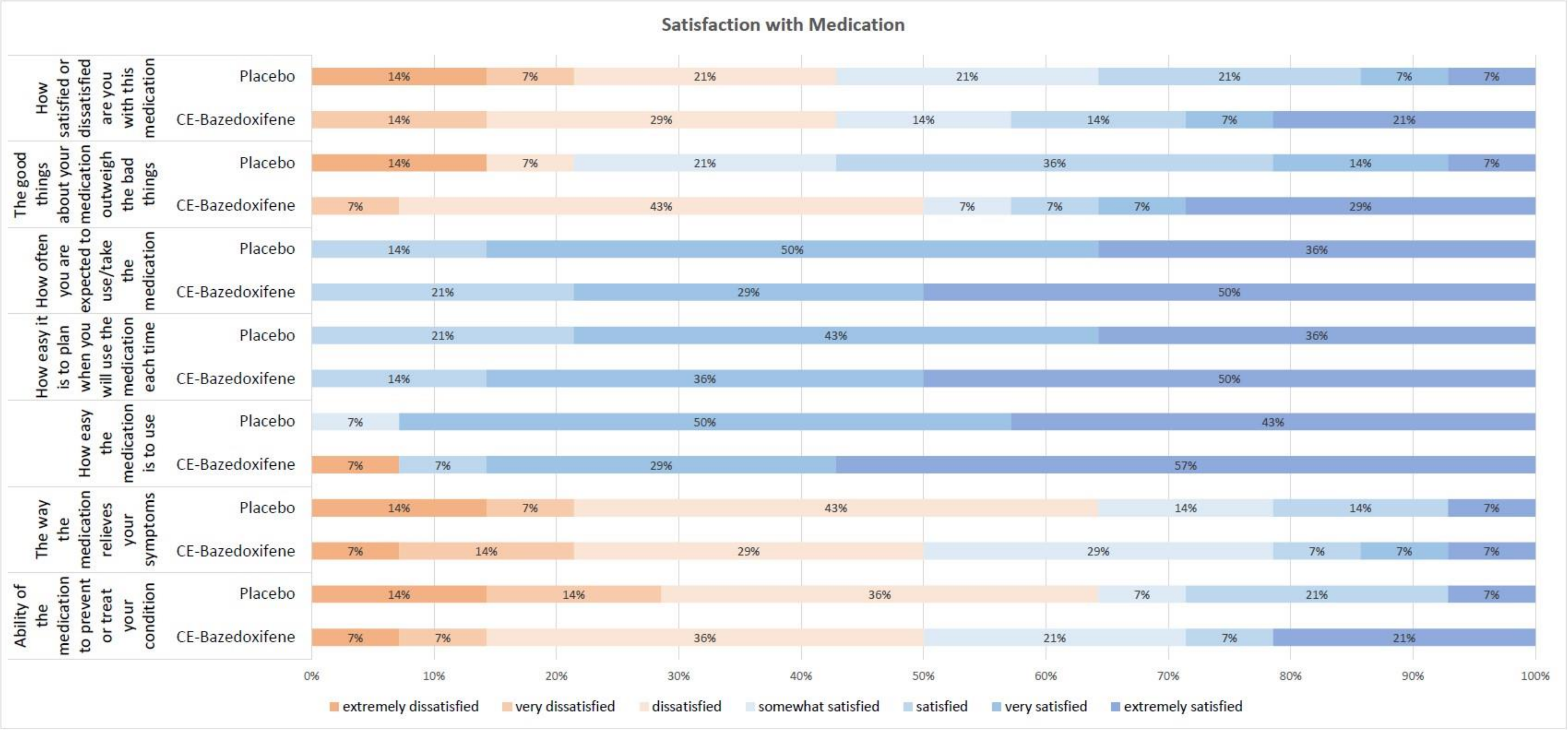

Supplementary Figure 5. Satisfaction with medication responses, by treatment arm

1 randomised participant = 7%

CE-Bazedoxifene, Conjugated Estrogens-Bazedoxifene

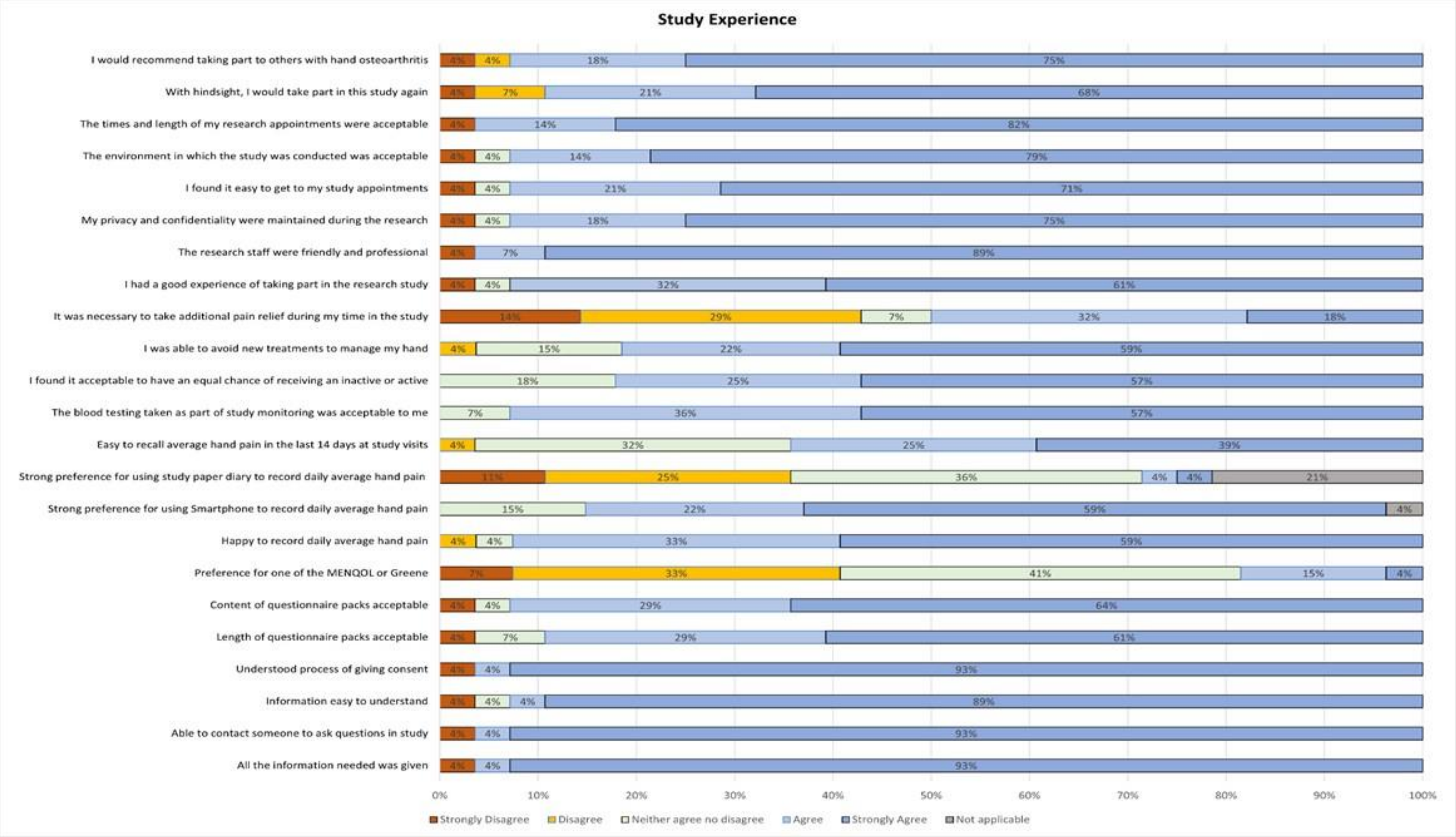

Supplementary Figure 6. End of study experience questionnaire
Results are not stratified by treatment arm. 1 randomised participant = 3.5%. MENQOL, Menopause specific Quality of Life questionnaire

Supplementary methods: Focus Groups

Aim

The aim was to explore the acceptability of the HOPE-e study from the perspective of people participating in the study.

Ethical approvals

Though covered broadly in the initial study protocol, Amendment 8 supported this work.

Qualitative methods

Topic areas in the semi-structured interview guide included why participants participated in HOPE-e, whether there was anything they would change about the trial, trial processes, procedures and practicalities, outcome measures; the impact of SARS-CoV-2 upon participation, ease of medication use, acceptability and thoughts about Hormone Replacement Therapy, allocation concealment and views regarding outcome; what advice they would give to someone deciding whether to take part in HOPE-e and anything else they would like the study team to know about HOPE-e.

Purposive sampling was used to achieve a sample which included people from both trial arms (and both methods of remote hand pain rating), of varying age, symptom severity, types of joints affected and differing global impressions of change, across the three study sites, see supplementary table 18.

Data Collection

Two on-line (Microsoft Teams) focus groups (total=10 participants) were held and recorded (duration for group one = 1 hour 28 minutes, group two = 1 hour 5 minutes), facilitated by CML, with additional observer AF.

Data Analyses

Recordings were transcribed (CML). Since the interview guide was based around, and used to inform about identified topics, a codebook approach to thematic analyses (TA) was used (CML and secondary review by other study team members, JW/AF) (Braun, Clarke, 2021).

Considerations for a full trial

Other study members (JW/AF/FEW) were involved in reviewing with CML and summarising suggestions from this work for consideration to inform a main trial arising from the focus group analysis (Supplementary Table 18).

Variable	Parameter 1 (n)	Parameter 2 (n)	Parameter 3 (n)
Treatment arm	CE-Bazedoxifene (6)	Placebo (4)	
Patient Global Impression of Change	Improved (4)	Worse (3)	No change (3)
Study Site	NOC (4)	CXH (1)	WHMP (5)
Baseline hand function (FIHOA)	Good, FIHOA Score 0-10 (8)	Moderate to Poor, FIHOA Score 11-20 (2)	Poor, FIHOA Score 21-30 (0) [1]
Age category	50-55 years (2)[2]	56-60 years (4)	61-66 years (4)
Study medication adherence	Per protocol (10)	Withdrawn (0)[1]	Withdrawn during weaning period (0) [1]
Joints affected	IPJ and BofT (6)	IPJ only (4)	BofT only (0) [1]

Supplementary Table 18. Sampling frame defining the variables of the participants who participated in the two focus groups

Seven variables were pre-defined to aid selection of participants for the focus groups. Where possible, proportionate numbers for each parameter were selected to take part in the focus groups. n = number of participants for each variable across the two focus groups.

[1] Only 1-2 participants from the overall study group were within this parameter
[2] Although the age range for the study was 40-65, no-one under the age of 50 was randomised in the study and therefore those aged 40-49 were not included in the sampling frame
CE-Bazedoxifene, Conjugated Estrogens-Bazedoxifene; NOC, Nuffield Orthopaedic Centre, Oxford University Hospitals NHS Foundation Trust, Oxford, UK; CXH, Charing Cross Hospital, Imperial College Healthcare NHS Trust, London, UK; WHMP, White Horse Medical Practice, Oxfordshire, UK; FIHOA, Functional Index for Hand Osteoarthritis; IPJ, Interphalangeal joints; BofT, Base of Thumb.

Supplementary results & limitations: Focus Groups

From 45 early codes, three main categories were developed: I. Participating in the HOPE-e study. II. Views around the intervention. III. Experience of Hand OA.

I**. Participating in the HOPE-e study.** Generally participants found study processes acceptable, liked the study team and appreciated their expertise and interest, and would advise others to take part in HOPE-e. Identified issues surrounding the trial are summarised below

II. Views around the intervention. Ahead of the focus groups there had been database lock and participants were intentionally informed of their allocation, ahead of taking part to enable this discussion (for all participants this was after their final data collection at Week 28). Intervention versus placebo and perceived outcome: Many participants were surprised or 'disappointed' (Group 2 pt 6) when their group allocation was revealed to them, having believed they were in a different treatment arm. This was especially so if they had assumed they had received the placebo because they thought their symptoms had not improved. Participants recognised the bad publicity and controversy about taking hormone replacement therapy (HRT) in the past and the concern about HRT causing cancer and found discussing this with the lead investigator reassuring for them. Participants described clear engagement with taking their study medication and gave examples of many strategies they used to remember to take their tablets. Some participants realised they had noticed symptoms increasing after stopping taking what they had found to be active drug and this has been raised as an issue.

III. Experience of Hand OA. I'm old before my time: Participants in group 1 described their hand OA as prematurely aging. Impact on daily life: Participants described the significant limiting and sometime humiliating impact hand OA has upon their lives and daily activities such as opening food packaging and lifting objects like vases and flour. Fear of provoking pain also impacted upon activities:
'*I can't turn a key in a door, pulling up my tights, it's everything. Putting boots on, pulling boots on, …It's not that I can't do it, I guess it's the fear of the pain when you grip something* (*Group 1 pt 4).*
It could be hard to explain the impact of hand OA to family and friends and participants felt there was a lack of awareness about hand OA. Some participants spoke of their concerns about the future and the need to live life to the full now (including changing job) while they could because '*I'm thinking in 5, 10 years time I'm not going to be able to do them'* (Group 1 pt 4). Many participants spoke about their family history of hand OA, with children, siblings (including a brother), mothers and grandmothers having the condition and about risk factors of developing hand OA. Several participants in group 1 wondered whether there was a link between hand OA and hypermobility as they, and relatives, had both conditions. Participants in both groups recounted problematic consultations with their General Practitioners, '*he was very, I don't know, disinterested, unsupportive, just like 'yes you have' and it was almost like a shrug of the shoulders and I thought that was really kind of disappointing (Group 2 pt 4).* This negative experience was repeated by many group members '*Where do you start? I, from my GP too I got the 'there's nothing we can do, you know that's just it'* (*Group 2 pt 3*). I'd have better treatment if Hand OA and the menopause affected men: Participants in both groups believed that there was a lack of research into the menopause and hand OA because these affect women rather than men. There was a stated belief that, if men had these conditions there would be a body of existing evidence and available treatments that do not exist at the present time; ***'****maybe I'm sexist but I just think if this had happened to men would something have happened sooner in terms of research and treatments and things like that but maybe I'm biased because I'm female (Group 2 pt 1)'.* Participants also thought women had accepted these conditions as a necessary part of aging but that it was time to change and be proactive in their efforts to obtain treatments. Generally, participants believed there was a gender issue surrounding the attitude towards, and treatments available for the menopause and hand OA.

Limitations when considering focus group results: There were some limitations to the design of the focus groups. Participants were post-menopausal women who had agreed to take part in HOPE-e, so arguably were open/supportive of considering taking HRT for hand OA. The study team did not have consent to approach people who had declined to participate in HOPE-e and these people may have/are likely to hold different views than those provided in the focus groups. Obtaining views about taking HRT for hand OA from people deciding not to participate would be needed to further inform the acceptability of the intervention.
The focus groups took place well after the trial had finished for some participants. The richness of the data provided in groups indicated that people had remembered views and experiences important to them, but there was some evidence that some areas were remembered less clearly. A further limitation is that participants in this feasibility study did not demonstrate wide diversity and further research would be needed to explore the acceptability of the intervention in other areas and communities within the UK.

Issue	Suggestion
Difficulty opening study medication packaging	Explore aids and/or provide simple advice to assist this process and make available to people if they have difficulties/pain opening study drugs
End of trial 'that's it'	To consider a peer group/group discussion after the end of the trial
Fidelity	To ensure all participants are given the same information (including general advice), and at the same timepoint, in the study
Information sheets	Participants were from a narrow demographic background, the information (level, language, tone, etc) would need to be explored with a diverse group of people to ensure they are appropriate.
Lack of diversity amongst study participants	Work will be needed to assess the acceptability of the intervention in other communities, including people from ethnic minority groups, people from lower education and socio-economic backgrounds
Menopause questionnaire found depressing by n=1	This might suggest that the shorter questionnaire may be more appropriate when the team decides which one of the two questionnaires to use in a main study.
Numerical rating scales (NRS)<br>- rate both hands / dominant and non dominant<br>- consider other NRS<br>- difficulty rating pain, and rating their pain against their peers	To consider rating both hands, separately to consider adding an NRS for stiffness (and possibly muscle weakness).<br>To improve the instructions for the NRS to make it clearer that individual change scores are being measured.<br>To explore with people whether they would prefer rating their pain continuously throughout the study, rather than around assessment points, to show how pain over time varies.
Participants thought having a female trial team who were invested in the trial was important	In a main trial, where trial teams may not all be female, then training may be necessary so that team members reassure participants that they are as invested in the trial as the lead investigator and that they understand and appreciate women's health issues.
People's views varied about visit type – in-person, telephone, on-line	There was agreement that the first and final visit should be in-person, but that the team should consider some flexibility in visit types for interim visits.
Photographs of hands caused distress for some	To consider if/how often to have photographs taken and how to support people finding this upsetting, to explore whether people would find this easier/more acceptable if photographs are taken in clinic versus at home.
Recording pain – some people felt activities influence pain more than medication	Activities that make the pain better/worse may influence pain recording more than the intervention, consider whether to capture this.
Several participants reported adverse symptoms increasing after stopping the intervention	To consider a longer follow up period to measure symptoms after medication has stopped, to explore flare up or deterioration after stopping the drug.
Strategies used to remember to take study medication	The strategies from participants, in their own words, could be supplied with the first study medication to promote engagement.
Stress can adversely affect conditions	To consider an outcome measure for stress, via a NRS or a question included in the questionnaire.
The appearance of the medication mattered	The medication in both groups needs to look the same in a future study.
Sample in this study were participants of HOPE-e	If a main trial takes place, this will need to include interviews with people deciding not to take part, to explore their views about HRT.

Supplementary Table 19. Recommendations from the focus group analysis for consideration in design of a full trial

NRS, numerical rating scale; HRT, hormone replacement therapy

CONSORT 2010 checklist of information to include when reporting a pilot or feasibility trial

Section/Topic	Item No	Checklist item	Reported on page No
**Title and abstract**			
	1a	Identification as a pilot or feasibility randomised trial in the title	1
	1b	Structured summary of pilot trial design, methods, results, and conclusions (for specific guidance see CONSORT abstract extension for pilot trials)	6
**Introduction**			
Background and objectives	2a	Scientific background and explanation of rationale for future definitive trial, and reasons for randomised pilot trial	7-8
	2b	Specific objectives or research questions for pilot trial	7-8
**Methods**			
Trial design	3a	Description of pilot trial design (such as parallel, factorial) including allocation ratio	9
	3b	Important changes to methods after pilot trial commencement (such as eligibility criteria), with reasons	Appendix, p2
Participants	4a	Eligibility criteria for participants	9 and appendix p5
	4b	Settings and locations where the data were collected	9 and appendix p10
	4c	How participants were identified and consented	9
Interventions	5	The interventions for each group with sufficient details to allow replication, including how and when they were actually administered	10
Outcomes	6a	Completely defined prespecified assessments or measurements to address each pilot trial objective specified in 2b, including how and when they were assessed	10-11 and appendix p7
	6b	Any changes to pilot trial assessments or measurements after the pilot trial commenced, with reasons	12, 14 and appendix p2
	6c	If applicable, prespecified criteria used to judge whether, or how, to proceed with future definitive trial	12
Sample size	7a	Rationale for numbers in the pilot trial	12
	7b	When applicable, explanation of any interim analyses and stopping guidelines	N/A
Randomisation:			
Sequence generation	8a	Method used to generate the random allocation sequence	10
	8b	Type of randomisation(s); details of any restriction (such as blocking and block size)	10
Allocation concealment mechanism	9	Mechanism used to implement the random allocation sequence (such as sequentially numbered containers), describing any steps taken to conceal the sequence until interventions were assigned	10
Implementation	10	Who generated the random allocation sequence, who enrolled participants, and who assigned participants to interventions	10
Blinding	11a	If done, who was blinded after assignment to interventions (for example, participants, care providers, those assessing outcomes) and how	10
	11b	If relevant, description of the similarity of interventions	10
Statistical methods	12	Methods used to address each pilot trial objective whether qualitative or quantitative	12-13 and appendix p30
**Results**			
Participant flow (a diagram is strongly recommended)	13a	For each group, the numbers of participants who were approached and/or assessed for eligibility, randomly assigned, received intended treatment, and were assessed for each objective	14 and Figure 1
	13b	For each group, losses and exclusions after randomisation, together with reasons	14, Figure 1, Table 2 and appendix p13

Recruitment	14a	Dates defining the periods of recruitment and follow-up	Figure 1
	14b	Why the pilot trial ended or was stopped	N/A
Baseline data	15	A table showing baseline demographic and clinical characteristics for each group	Table 2
Numbers analysed	16	For each objective, number of participants (denominator) included in each analysis. If relevant, these numbers should be by randomised group	Table 3, Appendix p11-27
Outcomes and estimation	17	For each objective, results including expressions of uncertainty (such as 95% confidence interval) for any estimates. If relevant, these results should be by randomised group	Figure 2 and 3, Appendix p11-12, 20-25
Ancillary analyses	18	Results of any other analyses performed that could be used to inform the future definitive trial	Appendix p31
Harms	19	All important harms or unintended effects in each group (for specific guidance see CONSORT for harms)	Table 3
	19a	If relevant, other important unintended consequences	N/A
**Discussion**			
Limitations	20	Pilot trial limitations, addressing sources of potential bias and remaining uncertainty about feasibility	21
Generalisability	21	Generalisability (applicability) of pilot trial methods and findings to future definitive trial and other studies	21
Interpretation	22	Interpretation consistent with pilot trial objectives and findings, balancing potential benefits and harms, and considering other relevant evidence	18-22
	22a	Implications for progression from pilot to future definitive trial, including any proposed amendments	19-22
**Other information**			
Registration	23	Registration number for pilot trial and name of trial registry	13
Protocol	24	Where the pilot trial protocol can be accessed, if available	9
Funding	25	Sources of funding and other support (such as supply of drugs), role of funders	27
	26	Ethical approval or approval by research review committee, confirmed with reference number	9

Supplementary Table 20. CONSORT, Feasibility extension checklist

References

Scoring the MHQ | themhq. Available: https://mchoirresearch.wixsite.com/themhq/scoring-the-mhq. Accessed Aug 18 2021.

Altman, R., Alarcon, G., Appelrouth, D., et al. 1990, "The American College of Rheumatology criteria for the classification and reporting of osteoarthritis of the hand", *Arthritis and rheumatism,* vol. 33, no. 11, pp. 1601-1610.

Bang, H., Ni, L. & Davis, C.E. 2004, "Assessment of blinding in clinical trials", *Controlled clinical trials,* vol. 25, no. 2, pp. 143-156.

Braun, V. & Clarke, V. 2021, *Thematic analysis: a practical guide,* Sage Publications Ltd.

Burbos, N. & Morris, E.P. 2010, *Menopausal symptoms*. BMJ Clinical Evidence [Internet]. Available from: /pmc/articles/PMC3275139/. Accessed Oct 1 2021.

Chen Jiefeng 2008, *BLINDING: Stata module to compute blinding indexes*. Available: https://ideas.repec.org/c/boc/bocode/s456898.html. Accessed Oct 1 2021.

Chung, K.C., Pillsbury, M.S., Walters, M.R., Hayward, R.A. & Arbor, A. 1998, "Reliability and validity testing of the Michigan Hand Outcomes Questionnaire", *The Journal of Hand Surgery,* vol. 23, no. 4, pp. 575-87.

Dreiser, R.L., Maheu, E., Guillou, G.B., Caspard, H. & Grouin, J.M. 1995, "Validation of an algofunctional index for osteoarthritis of the hand", *Rev Rheum (Engl ed.),* vol. 62, no. 6 Suppl 1, pp. 43S-53S.

Greene, J.G. 1998, *Constructing a standard climacteric scale*, Maturitas 1998; 29: 25.

Janssen, M.F., Pickard, A.S., Golicki, D., Gudex, C., Niewada, M., Scalone, L., Swinburn, P. & Busschbach, J. 2013, "Measurement properties of the EQ-5D-5L compared to the EQ-5D-3L across eight patient groups: a multi-country study", *Quality of Life Research,* vol. 22, no. 7, pp. 1717-1727.

Lewis, J.E., Hilditch, J.R. & Wong, C.J. 2005, "Further psychometric property development of the Menopause-Specific Quality of Life questionnaire and development of a modified version, MENQOL-Intervention questionnaire", *Maturitas,* vol. 50, no. 3, pp. 209-221.

Marian, I.R., Goff, M., Williams, J.A.E., et al. 2021, "Hand Osteoarthritis: investigating Pain Effects of estrogen-containing therapy (HOPE-e): a protocol for a feasibility randomised placebo-controlled trial", *Pilot and feasibility studies,* vol. 7, no. 1, pp. 133.

Mathiowetz, V., Weber, K., Volland, G. & Kashman, N. 1984, *Reliability and validity of grip and pinch strength evaluations*, Elsevier BV.

Radtke, J., Terhorst, L. & Cohen, S. 2011, "The Menopause-Specific Quality of Life Questionnaire: psychometric evaluation among breast cancer survivors", *Menopause (New York, N.Y.),* vol. 18, no. 3, pp. 289-295.

van Hout, B., PhD, Janssen, M.F., PhD, Feng, Y., PhD, et al 2012, "Interim Scoring for the EQ-5D-5L: Mapping the EQ-5D-5L to EQ-5D-3L Value Sets", *Value in Health,* vol. 15, no. 5, pp. 708-715.
